# c-Maf-positive spinal cord neurons are critical elements of a dorsal horn circuit for mechanical hypersensitivity in neuropathy

**DOI:** 10.1016/j.celrep.2023.112295

**Published:** 2023-03-21

**Authors:** Noémie Frezel, Matteo Ranucci, Edmund Foster, Hagen Wende, Pawel Pelczar, Raquel Mendes, Robert P. Ganley, Karolina Werynska, Simon d’Aquin, Camilla Beccarini, Carmen Birchmeier, Hanns Ulrich Zeilhofer, Hendrik Wildner

**Affiliations:** 1 Institute of Pharmacology and Toxicology, University of Zürich, 8057 Zürich, Switzerland; 2 Max Delbrück Center, 13125 Berlin, Germany; 3 Center for Transgenic Models (CTM), University of Basel, 4001 Basel, Switzerland; 4 Pharmaceutical Sciences, Swiss Federal Institute of Technology (ETH) Zürich, 8092 Zürich, Switzerland

**Keywords:** spinal cord,, corticospinal tract,, neuropathic pain,, deep dorsal horn,, allodynia,, allokinesis, protein kinase C gamma, spinal interneurons, nociception

## Abstract

Corticospinal tract (CST) neurons innervate the deep spinal dorsal horn to sustain chronic neuropathic pain. The majority of neurons targeted by the CST are interneurons expressing the transcription factor c-Maf. Here, we used intersectional genetics to decipher the function of these neurons in dorsal horn sensory circuits. We find that excitatory c-Maf (c-Maf^EX^) neurons receive sensory input mainly from myelinated fibers and target deep dorsal horn parabrachial projection neurons and superficial dorsal horn neurons, thereby connecting non-nociceptive input to nociceptive output structures. Silencing c-Maf^EX^ neurons has little effect in healthy mice but alleviates mechanical hypersensitivity in neuropathic mice. c-Maf^EX^ neurons also receive input from inhibitory c-Maf and parvalbumin neurons, and compromising inhibition by these neurons caused mechanical hypersensitivity and spontaneous aversive behaviors reminiscent of c-Maf^EX^ neuron activation. Our study identifies c-Maf^EX^ neurons as normally silent second-order nociceptors that become engaged in pathological pain signaling upon loss of inhibitory control.

## Highlights

• Silencing of c-Maf^EX^ neurons has no impact on acute pain, while their activation induces pain
• Silencing of c-Maf^EX^ neurons in neuropathic mice reduces hypersensitivity and allodynia
• Inhibitory parvalbumin neurons silence c-Maf^EX^ neurons in naive mice
• c-Maf^EX^ neurons can directly activate deep dorsal horn projection neurons

## Graphical abstract

**Figure:**
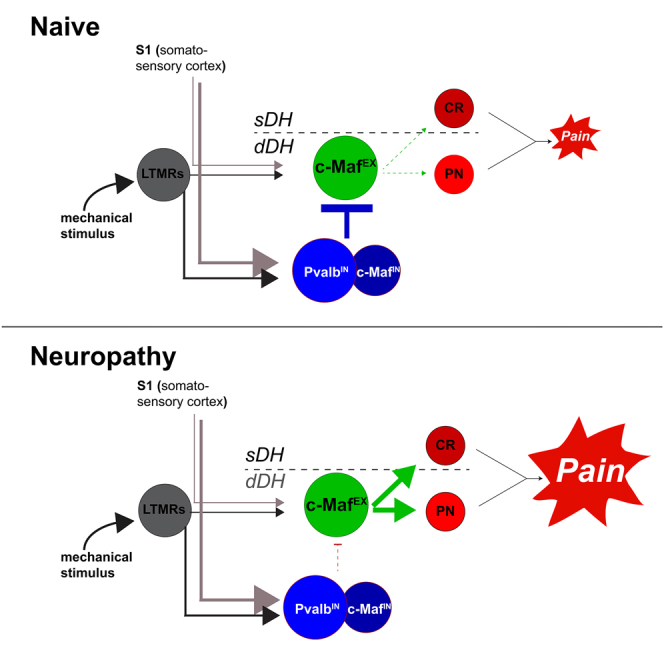

## Teaser

Frezel et al. use intersectional genetics to identify a population of deep dorsal horn excitatory neurons that act as silent second-order nociceptors that are only recruited into pain pathways after nerve injury. They can engage nociceptive pathways via the superficial dorsal horn or via deep dorsal horn projection neurons.

## Introduction

Patients suffering from chronic pain not only display increased sensitivity to noxious stimuli but often also perceive innocuous stimuli (e.g., touch) as painful. This phenomenon is known as allodynia. A wide variety of alterations potentially contributing to allodynia have been proposed, ranging from changes in peripheral neurons and spinal neurons to changes at supraspinal sites.^1^^,^^2^^,^^3^^,^^4^^,^^5^ Recently, it has been described that the corticospinal tract (CST), which in naive mice is regarded as an important element for top-down control of voluntary movement,^6^^,^^7^ critically contributes to mechanical allodynia in nerve-injury-induced chronic pain states.^8^ In these conditions innocuous low-threshold afferent input is thought to gain access to superficial dorsal horn nociceptive specific circuits via polysynaptic pathways, thus producing touch-evoked allodynia.^9^^,^^10^^,^^11^^,^^12^ Liu et al.^8^ suggested that CST neurons located in the somatosensory cortex (S1) synapse onto neurons in the deep dorsal spinal horn, an area also termed “low-threshold mechanoreceptor (LTMR) recipient zone,” as it receives input predominantly from myelinated sensory afferents conveying innocuous information about touch and proprioception.^7^^,^^13^ Taken together, these findings suggest that deep dorsal horn neurons that integrate low-threshold primary afferent input and descending information from S1 are critical components of the neural circuitry that controls mechanical pain perception after nerve injury. While several deep dorsal horn interneuron populations have been identified that receive corticospinal and low-threshold peripheral input,^13^ it is only incompletely understood which of them are required for mechanically evoked pain after nerve injury.

We have previously identified dorsal horn neurons expressing the transcription factor c-Maf as a main target population of CST neurons in S1.^14^ Here, we have employed intersectional virus-based strategies for circuit tracing and functional manipulation to identify excitatory c-Maf neurons as critical elements of a spinal circuit involved in the generation of nerve-injury-induced mechanical allodynia.

## Results

### c-Maf is expressed in subsets of deep dorsal horn excitatory and inhibitory interneurons

**Figure 1:**
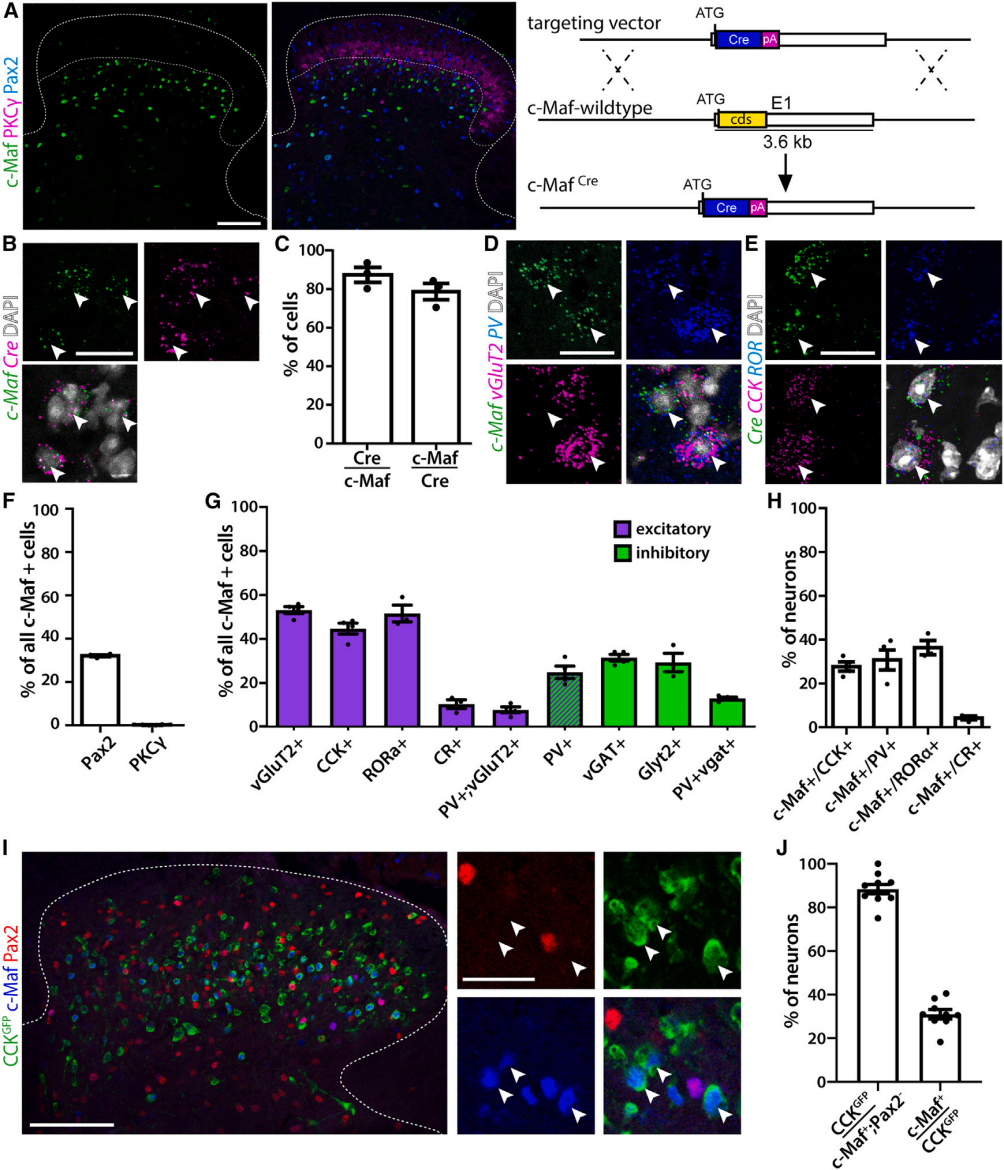
c-Maf is expressed in subsets of deep dorsal horn interneurons (A) c-Maf expression in the dorsal spinal cord and schematic representation of the generation of the c-Maf^Cre^ allele. (B) Double *in situ* hybridization showing the overlap between *c-Maf* and *Cre* mRNAs in the c-Maf^Cre^ mouse spinal cord. (C) Quantification of (B) (n = 3, 605 neurons). (D) Triple *in situ* hybridization showing overlap between *c-Maf-*, *VGlut2*-, and *PV*-expressing neurons. (E) Triple *in situ* hybridization showing overlap between *Cre*^+^ (c-Maf)-, *CCK*-, and *RORα*-expressing neurons. (F) Quantification of the co-expression of c-Maf, Pax2, and PKCγ proteins as depicted in (A) (n = 4, 1,293 c-Maf^+^ neurons). (G) Quantification of the proportion of c-Maf neurons expressing other markers of deep dorsal horn neurons (*VGlut2*: n = 4, 1,045 *c-Maf*^+^ neurons; *CCK*: n = 4, 848 *c-Maf*^+^ neurons; *RORα*: n = 3, 317 *c-Maf*^+^ neurons; *CR*: n = 3, 302 *c-Maf*^+^ neurons; *PV*: n = 4, 878 *c-Maf*^+^ neurons; *vGAT*: n = 4, 878 *c-Maf*^+^ neurons; *Glyt2*: n = 3, 302 *c-Maf*^+^ neurons). Magenta bars represent genes exclusively/predominantly expressed in excitatory neurons, while green bars represent co-expression with inhibitory marker genes. (H) Quantification of the proportion of *CCK*^+^, *PV*^+^*RORα*^+^, and *CR*^+^ neurons expressing *c-Maf* (n = 4, 1,362 *CCK*^+^ and 513 *PV*^+^ neurons; n = 3, 734 *CR*^+^ and 454 *RORα*^+^ neurons). (I) Co-labeling of GFP, Pax2, and c-Maf in the dorsal spinal cord of CCK^GFP^ (CCK^Cre^;ROSA26^fls-NuTRAP^) animals. Arrows indicate excitatory c-Maf neurons (c-Maf^+^;Pax2^−^) co-expressing GFP. (J) Quantification of (I) (n = 3, 309 neurons). Note that the vast majority of excitatory c-Maf neurons (c-Maf^+^;Pax2^−^) co-express GFP while only one-third of the CCK^GFP^ neurons co-express c-Maf. Error bars denote ±SEM. Scale bars, 100 μm (A and I) and 20 μm (B, D, and E).

To address the role of c-Maf neurons in dorsal horn neural circuits, we generated a c-Maf^Cre^ knockin mouse line (Figure 1A). Eutrophic expression of *Cre* was verified using multiplex *in situ* hybridization on spinal cord sections of adult c-Maf^Cre^ mice (Figure 1B). We found that 87.4% ± 3.9% of *c-Maf*^+^ neurons co-expressed *Cre* mRNA and detected *c-Maf* mRNA in 78.8% ± 4.2% of *Cre*^+^ neurons (Figure 1C). We next analyzed the localization and molecular identity of spinal c-Maf/Cre neurons using immunohistochemistry and multiplex *in situ* hybridization. Neurons expressing c-Maf were present in laminae III and IV (Figure 1A), ventral to the protein kinase C γ (PKCγ) plexus, which delineates the border between the superficial and deep dorsal horn.^15^ Only few c-Maf neurons (0.18% ± 0.1%) also expressed PKCγ (Figures 1A and 1F). To further characterize c-Maf neurons, we used *c-Maf* or *Cre* probes together with probes for a variety of previously reported marker genes for dorsal horn neurons^16^^,^^17^^,^^18^^,^^19^^,^^20^ (Figures 1D–1H and S1). Consistent with a previous report,^21^ we found that more than half of the c-Maf neurons were excitatory (52.9% ± 1.6% expressed *VGLUT2*, Figures 1D and 1G) and one-third were inhibitory (32.1% ± 0.4% expressed Pax2, 31.3% ± 1.6% expressed *VGAT*, and 29.1% ± 4.2% expressed *Glyt2*, Figures 1A, 1F, 1G, and S1). Single-cell profiling experiments^16^^,^^22^ have suggested *c-Maf* as a marker for a molecular defined family of excitatory dorsal horn neurons present in 2 out of 15 excitatory (Glut1 and Glut2) and 3 out of 15 inhibitory (GABA11, GABA12, and GABA13) neuronal subpopulations. Consistent with the single-cell data, we found that 84% of the excitatory c-Maf neurons (44.5% ± 2.5% of all c-Maf neurons) expressed *cholecystokinin* (*CCK*) (Figures 1E and 1G), 80% (51.3% ± 3.8% of all c-Maf neurons) expressed *RORα* (Figures 1E, 1G, and S1C), and 24.7% ± 2.8% of all c-Maf neurons expressed *parvalbumin* (*PV*) (Figures 1D, 1G, and S1A). Again, in line with single-cell data, *PV* was found to be expressed in both excitatory and inhibitory c-Maf neurons (7.6% ± 1.3% of the excitatory and 12.7% ± 0.6% of the inhibitory c-Maf neurons). Only few c-Maf neurons were calretinin (CR) positive (10.2% ± 2.0% of all c-Maf cells) (Figures 1G, S1B, and S1D). Vice versa, we also determined the percentages of c-Maf neurons among the *CCK*, *RORα*, *CR*, and *PV* populations and found that 27.7% ± 2.1% of *CCK*-positive neurons expressed *c-Maf*, 36.4% ± 3.2% of *RORα*-positive neurons, and 30.7% ± 4.6% of *PV*^+^ neurons, and again only a small overlap with *CR* neurons was observed (4.23% ± 0.9% of *CR*^+^ neurons, Figures 1H and S1D). Our data thus indicate that excitatory c-Maf neurons constitute a subset of the larger CCK population. To further verify this finding, we labeled spinal CCK cells by crossing CCK^Cre^ mice to a reporter line (NuTRAP) that expresses GFP in a Cre-dependent manner (labeled cells are termed CCK^GFP^). In subsequent co-labeling experiments, we co-stained spinal cord sections with antibodies against GFP, c-Maf, and Pax2 (Figure 1I). In line with our previous results, we found that 88.4% ± 2.2% of all excitatory c-Maf neurons (c-Maf^+^; Pax2^−^) co-expressed GFP. Vice versa, about one-third of CCK^GFP^ cells (31% ± 2.1%) were c-Maf positive, while almost no inhibitory c-Maf neurons (c-Maf^+^; Pax2^+^) co-expressed GFP (Figures 1I and 1J). Taken together, our findings are consistent with single-cell RNA-sequencing data^16^ (http://linnarssonlab.org/dorsalhorn/) indicating c-Maf expression in a PKCγ-negative subfamily of CCK neurons that represents 2 out of 15 excitatory populations of spinal dorsal horn neurons (Glut1 and Glut2).

### Intersectional targeting strategies enable selective targeting of either excitatory or inhibitory c-Maf neurons

**Figure 2:**
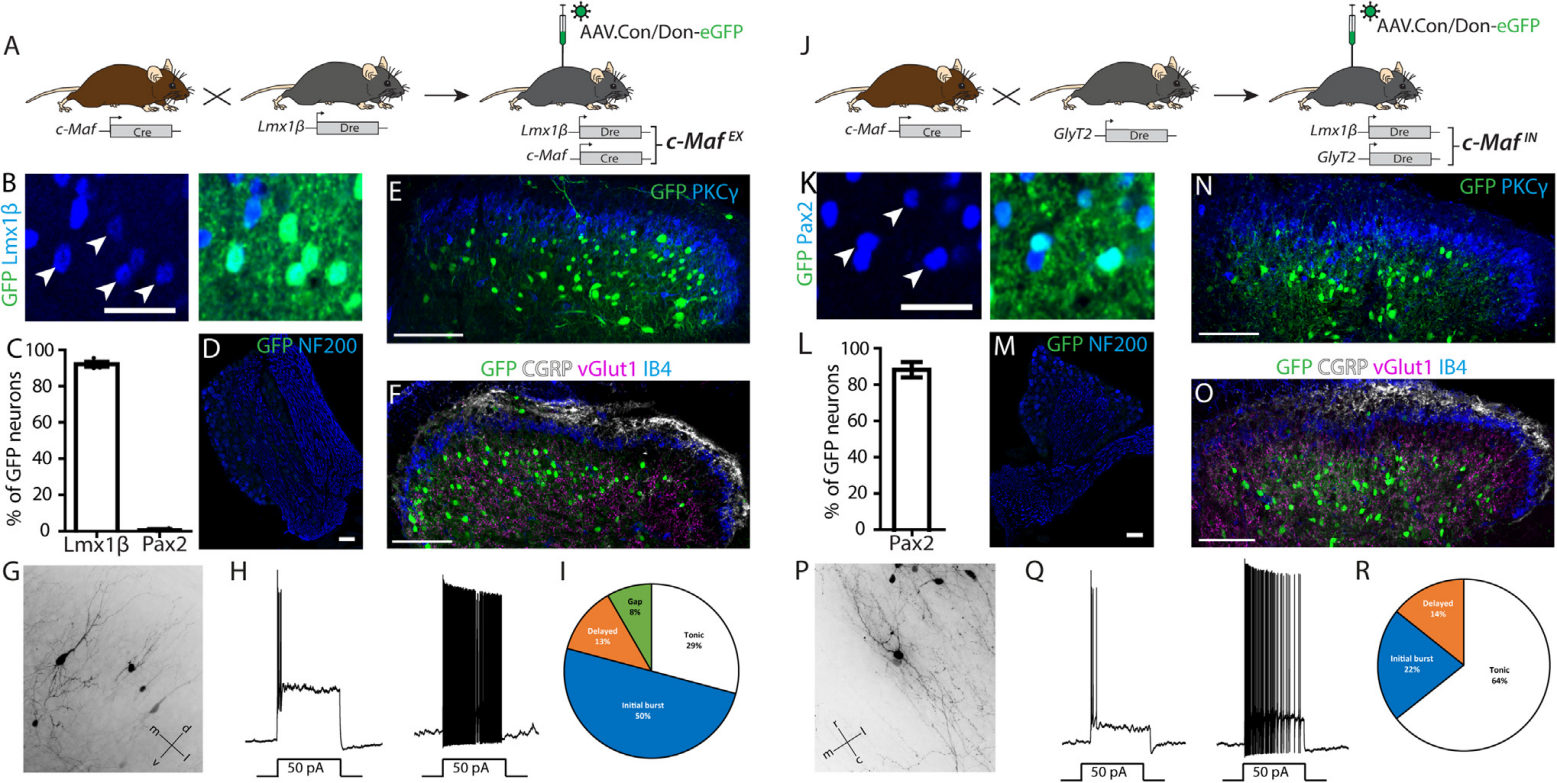
Intersectional targeting of c-Maf^EX^ and c-Maf^IN^ neurons (A) Intersectional targeting strategy of *c-MafEX* neurons. (B) Immunofluorescence staining of a transversal section of the lumbar spinal cord of *c-MafEX* mice injected in the spinal cord with rAAV9.CAG.C_on_/D_on_.eGFP, showing the overlap between eGFP^+^ and Lmx1b^+^ neurons. Scale bars, 50 μm. (C) Quantification of the number of eGFP^+^ neurons positive for Lmx1b and Pax2 in (B) (n = 4, 853 *eGFP*^+^ neurons). (D) Immunofluorescence staining of a lumbar DRG section of c-Maf^EX^ mice showing no expression of eGFP in sensory neurons following spinal cord injection. Scale bars, 50 μm. (E) Localization of eGFP-labeled neurons relative to PKCγ immunoreactive cell layer (n = 4). Scale bars, 100 μm. (F) Localization of eGFP-labeled neurons relative to CGRP, IB4, and VGlut1 immunoreactivity (n = 4). Scale bars, 100 μm. (G) Morphology of sparsely labeled c-Maf^EX^ neurons. Coordinates are dorsal and ventral (d + v) and medial and lateral (m + l). (H) Example traces recorded from c-Maf^EX^ neurons. (I) Venn diagram indicating the proportion of firing patterns that have been observed in c-Maf^EX^ neurons (n = 22 cells). (J) Intersectional targeting strategy of *c-MafIN* neurons. (K) Immunofluorescence staining of a transversal section of the lumbar spinal cord of *c-MafIN* mice injected in the spinal cord with rAAV9.CAG.C_on_/D_on_.eGFP, showing the overlap between eGFP^+^ and Pax2^+^ neurons. Scale bars, 50 μm. (L) Quantification of the number of eGFP^+^ neurons positive for pax2^+^ in (C) (n = 3, 611 neurons). (M) Immunofluorescence staining on DRG sections showing no expression of the eGFP transgene in sensory neurons. Scale bars, 50 μm. (N) Localization of eGFP-labeled neurons relative to PKCγ immunoreactive cell layer (n = 3). Scale bars, 100 μm. (O) Localization of eGFP-labeled neurons relative to CGRP, IB4, and VGlut1 immunoreactivity (n = 3). Scale bars, 100 μm. (P) Morphology of sparsely labeled c-Maf^IN^ neurons. Coordinates are rostral and caudal (r + c) and medial and lateral (m + l). (Q) Example traces recorded from c-Maf^IN^ neurons. (R) Venn diagram indicating the proportion of firing patterns that have been observed in c-Maf^IN^ neurons (n = 18 cells). Error bars denote ±SEM. Scale bars, 50 μm (B and K) and 100 μm (D–F and M–O).

As outlined above, c-Maf neurons constitute a mixed population of excitatory and inhibitory neurons, and c-Maf is also expressed in dorsal root ganglion (DRG) neurons.^23^ We therefore chose intersectional strategies to selectively target the excitatory or inhibitory c-Maf neuron family in the spinal cord. To this end, we crossed either a Lmx1β^Dre^ allele (Figure S2) or a GlyT2:Dre transgene^24^ into c-Maf^Cre^ mice, and employed Cre and Dre double-dependent reporter transgenes delivered via recombinant adeno-associated viruses (rAAVs) (Figures 2A and 2J). Lmx1β is expressed in the vast majority of dorsal horn excitatory neurons, whereas GlyT2 is a marker gene for inhibitory neurons of the deep dorsal horn. Neither gene is expressed in DRG neurons (Figure S2E).^25^^,^^26^ To validate our intersectional strategies, we injected the left lumbar spinal cord of c-Maf^Cre^;Lmx1β^Dre^ double transgenic mice (hereafter referred to as c-Maf^EX^ mice) and c-Maf^Cre^;GlyT2:Dre mice (c-Maf^IN^ mice) with an rAAV carrying a Cre/Dre double-dependent eGFP expression cassette (rAAV9.hEF1α.C_on_/D_on_-eGFP) (Figure 2A).

In c-Maf^EX^ mice, the vast majority of eGFP^+^ neurons were found in laminae III and IV of the dorsal horn and co-expressed Lmx1b, while the inhibitory marker Pax2 was virtually absent from eGFP^+^ neurons (Figures 2B–2F). As expected, DRG neurons were devoid of eGFP (Figure 2D). Comparable results were obtained when reporter mice were used instead of reporter viruses (Figure S3). Most c-Maf^EX^ (eGFP^+^) neurons were located ventral to the PKCγ plexus (Figure 2E). Only few eGFP^+^ cells also expressed PKCγ (1.47% ± 0.59% of eGFP^+^ neurons, Figures 2E and S3F). We used calcitonin gene-related peptide (CGRP) immunostaining and isolectin B4 (IB4) binding to label laminae I/II_o_ and II_i_, respectively,^27^ which together comprise the termination area of most nociceptive fibers.^1^^,^^28^ VGlut1 staining was used to label the LTMR recipient zone.^13^^,^^29^ Most eGFP^+^ neurons and their neuropil were located ventral to the IB4 and CGRP layers within the area of the VGlut1 axon terminals (Figure 2F) consistent with the distribution of c-Maf immunoreactivity (Figure 1A). In c-Maf^IN^ mice, the vast majority of eGFP^+^ neurons (88.7% ± 4.3%) were Pax2^+^ (Figures 2H and 2I). Again, no eGFP expression was detected in DRG neurons (Figure 2M). The localization of eGFP neurons (Figures 2N and 2O) was comparable with that found in c-Maf^EX^ neurons. Both c-Maf^EX^ and c-Maf^IN^ neurons were in the LTMR recipient zone, the area receiving low-threshold cutaneous and proprioceptive information,^13^^,^^29^^,^^30^ indicating that c-Maf neurons likely receive non-nociceptive LTMR sensory input.^7^^,^^8^^,^^13^^,^^17^^,^^31^^,^^32^ To further characterize the neurons targeted by our intersectional strategies, we analyzed their morphology and recorded basic biophysical parameters. Sparse labeling with an eGFP encoding replication-deficient rabies virus (see Albisetti et al.^33^) revealed that many c-Maf^EX^ neurons, especially in upper lamina III, displayed a vertical cell-like morphology with an apical dendrite extending toward the superficial laminae (Figure 2G) while the morphology of many c-Maf^EX^ neurons in deeper L III and L IV were less polarized (Figure S4A), more resembling central cells. These observations are consistent with morphologies that have been reported previously for deep dorsal horn RORα cells or CCK cells.^10^^,^^17^ Many c-Maf^IN^ neurons could be identified as islet cells (Figure 2P) or radial cells (Fig S4B). Next, we used c-Maf^Cre^;Lmx1β^Dre^/(GlyT2:Dre); Ai66 mice to identify and characterize the biophysical properties of c-Maf^EX^ (c-Maf^IN^) neurons. The majority of Maf^EX^ neurons displayed an initial burst firing pattern (50%). About one-third (29%) showed tonic firing, 13% delayed firing, and 8% gap firing (Figures 2H–2I). Maf^IN^ neurons predominantly presented a tonic firing pattern (64%) while some showed initial burst (22%) or delayed (14%) firing (Figures 2Q and 2R). Maf^EX^ and Maf^IN^ neurons had similar thresholds, resting membrane potentials, rheobase, action potential width, and input resistance but differed significantly in their average capacitance and after hyperpolarization (Figure S4C–S4I).

### c-Maf neurons integrate peripheral and supraspinal input

**Figure 3:**
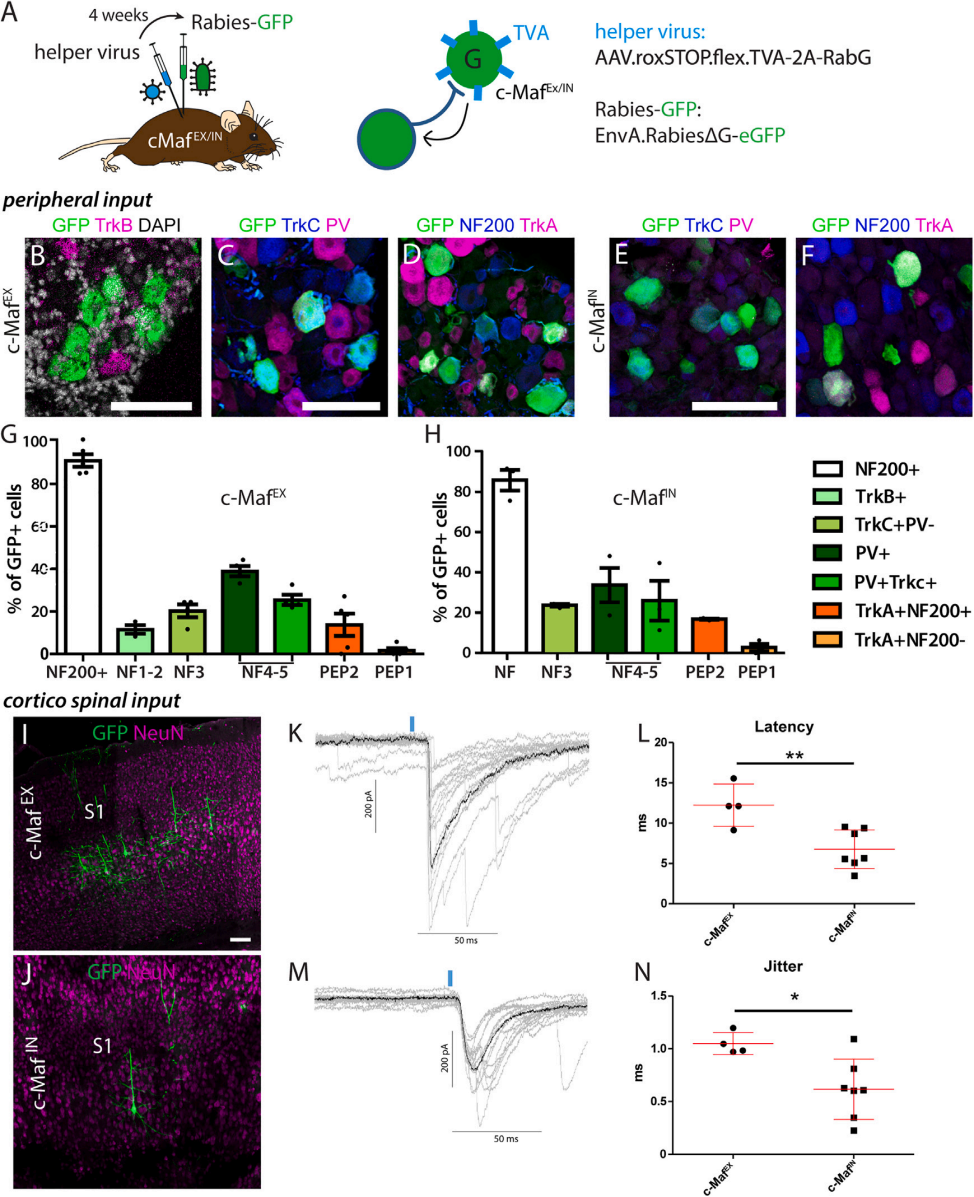
Retrograde rabies virus-based tracing of monosynaptic input to c-Maf neurons (A) A helper virus (TVA, RabG) was injected into the spinal cord of c-Maf^EX^ or c-Maf^IN^ mice, followed by injection of the EnvA-pseudotyped rabies virus (EnvA.RV.ΔG.eGFP). (B–F) RNAscope labeling or immunofluorescence staining on DRG sections showing overlap between GFP and markers of retrogradely traced sensory neurons in c-Maf^EX^ (B–D) or c-Maf^IN^ (E and F) mice. (G) Quantification of the number of GFP^+^ DRG neurons positive for NF200, TrkA (n = 5, 574 cells), PV and TrkC (n = 4, 349 cells), and *TrkB* (n = 3, 374 cells), in c-Maf^EX^ mice. (H) Quantification of the number of GFP^+^ DRG neurons positive for NF200, TrkA (n = 3, 239 cells), PV, and TrkC (n = 3, 167 cells) in c-Maf^IN^ mice. In (G) and (H), NF1–5 and PEP1–2 refer to the classification of sensory neurons proposed by Usoskin et al.:^35^ NF = NF200^+^, NF1–2 = TrkB^+^, NF3 = TrkC^+^;PV^−^, NF4–5 = PV^+^;(TrkC low), PEP2 = TrkA^+^;NF200^+^, PEP1 = TrkA^+^, NF200^−^. (I–N) (I and J) Immunofluorescence staining showing eGFP-labeled neurons in supraspinal sites retrogradely traced from c-Maf^EX^ (I–K) or c-Maf^IN^ (L–N) neurons. Neurons were found in the primary somatosensory cortex (CST neurons in layer 5 of S1, n = 4). (K–N) Slice recordings after optogenetic stimulation of ChR2-YFP expressing CST terminals in spinal cord slices (c-Maf^EX^ = 5 cells, c-Maf^IN^ = 7 cells). (K and M) Example traces recorded after optogenetic stimulation in c-Maf^EX^ or c-Maf^IN^ neurons. (L and N) Quantification of latencies and jitter recorded in c-Maf^EX^ or c-Maf^IN^ neurons. Error bars denote ±SEM. ^∗^p < 0.05, ^∗∗^p < 0.01 (unpaired Student’s t test). Scale bars, 100 μm.

To further characterize the nature of primary afferent input onto c-Maf^EX^ and c-Maf^IN^ neurons, we used monosynaptic rabies-based retrograde tracing^34^ (Figure 3A). A helper virus (rAAV.flex.rox.TVA.SAD19-G) was injected into the lumbar spinal cord of either c-Maf^EX^ or c-Maf^IN^ mice. This helper virus provided the TVA receptor gene permitting selective infection by EnvA-pseudotyped rabies virus, and in addition the rabies glycoprotein (SAD19-G) for *trans*-complementation to allow monosynaptic retrograde spread. Two weeks later, we injected a glycoprotein-deficient EnvA-pseudotyped rabies virus (EnvA.RV.ΔG.eGFP). To identify the subtypes of labeled sensory neurons in the DRG, we co-stained for known markers of sensory neuron types.^35^ Consistent with the localization of c-Maf^EX^ neurons in the termination area of LTMRs (Figure 2F), we found that the great majority of eGFP^+^ DRG neurons (91.0% ± 3.0%) were also positive for NF200, which marks myelinated DRG neurons (Figures 3D and 3G). Only few traced eGFP^+^ neurons expressed TrkA (13.8% ± 5.2% myelinated and 1.7% ± 1.1% unmyelinated TrkA^+^ neurons) (Figures 3D and 3G). As most primary afferent input onto c-Maf^EX^ neurons came from myelinated sensory afferents, we characterized these neurons in more detail. The majority of myelinated retrogradely labeled (eGFP^+^) DRG neurons expressed either PV (39.0% ± 2.4%), TrkC (20.3% ± 3.0%), or both markers (25.5% ± 2.4%) (Figures 3C and 3G). Further *in situ* hybridization experiments in sections of the DRG showed that 11.6% ± 2.0% of eGFP^+^ neurons expressed TrkB (Figures 3B and 3G). In contrast, we found that virtually no eGFP^+^ DRG neurons co-expressed markers of the non-peptidergic populations such as *Mrgpra3*, Plxnc1, or P2X3 (1.42% ± 0.5%, 1.75% ± 1.7%, and 8.14% ± 1.7%, respectively) (Figures S5A–S5D). Similar data were obtained for c-Maf^IN^ neurons. The vast majority of eGFP^+^ DRG neurons labeled from c-Maf^IN^ neurons were also positive for NF200 (86.3% ± 5.2%). eGFP^+^ neurons expressed PV (33.7% ± 8.6%), TrkC (23.6% ± 0.7%), or PV and TrkC (25.8% ± 10%), and some expressed TrkA (16.64% ± 0.4% TrkA^+^NF200^+^ and 2.38% ± 1.8% TrkA^+^NF200^−^) (Figures 3E, 3F, and 3H). Taken together, c-Maf^EX^ and c-Maf^IN^ neurons are located ventral to the CGRP and IB4 termination zone and are innervated mainly by non-nociceptive sensory neurons co-expressing NF200, PV, and TrkC, or NF200 and TrkB.

Since our previous data suggested monosynaptic connections between CST neurons of S1 and spinal c-Maf neurons,^14^ we examined eGFP expression also in supraspinal CNS areas. As expected, eGFP^+^ cells were present in the primary somatosensory cortex (S1, Figures 3I and 3J, n = 4) but also in the red nucleus (Figures S5E and S5G) and the rostroventral medulla (RVM, Figures S5F and S5H, n = 4), verifying that c-Maf^EX^ and c-Maf^IN^ neurons integrate descending supraspinal input with sensory input from different types of LTMRs and proprioceptors.

CST neurons innervating deep dorsal horn excitatory neurons have been reported to affect nociception in neuropathic mice by activating spinal excitatory CCK neurons.^8^ To verify functional connections between layer V S1 neurons and spinal c-Maf neurons as well as to investigate potential differences between CST innervation of c-Maf^EX^ or c-Maf^IN^ neurons, we performed slice recordings from the respective subtype. To this end, we overexpressed ChR2-YFP in CST neurons by injecting AAV.ChR2-YFP into S1 and recorded from labeled c-Maf^EX^ or c-Maf^IN^ neurons in the spinal cord sections of c-Maf^Cre^;Lmx1β^Dre^/(GlyT2:Dre);Ai66 mice after optogenetic stimulation of CST terminals. Latency and jitter recorded from c-Maf^EX^ neurons were significantly higher than those recorded from c-Maf^IN^ neurons (12.7 ± 1.1 ms vs. 6.8 ± 0.9 ms, respectively, p = 0.002) (Figures 3K–3N), suggesting that CST input is processed differentially by spinal c-Maf^EX^ or c-Maf^IN^ neurons.

### Behavioral effects of silencing of c-Maf^IN^ and c-Maf^EX^ neurons in healthy mice

**Figure 4:**
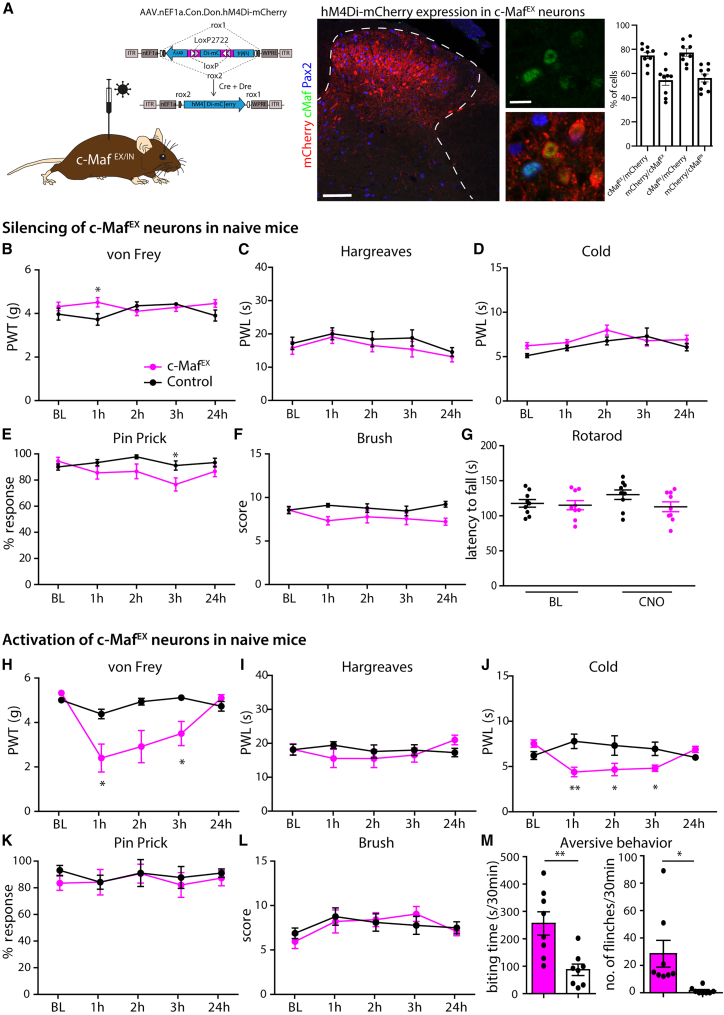
Pharmacogenetic silencing and activation of c-Maf^EX^ spinal interneurons in naive mice (A) Schematic illustration of an intersectional DREADD construct.^38^ DREADD expression (mCherry) was driven by the rAAV.EF1α.C_on_/D_on_.hM4Di-mCherry injected into the lumbar spinal cord of c-Maf^EX^ mice. Quantification of the percentage of DREADD-expressing cells that co-express c-Maf only (c-Maf^EX^) or c-Maf and Pax2 (c-Maf^IN^) after injection into c-Maf^EX^ or c-Maf^IN^ mice, respectively (n = 4 mice). Vice versa, the number of c-Maf^EX^ or c-Maf^IN^ cell that express the DREADD were quantified (n = 4 mice). (B–G) Behavioral responses after hM4Di-mediated silencing of c-Maf^EX^ neurons (hM4Di: c-Maf^EX^: n = 9; control: n = 9; Table S1). Responses to mechanical stimulation (B, E, F), heat (C), cold (D), and motor coordination assessed with the rotarod (G). (H–M) Behavioral responses after hM3Dq-mediated activation of c-Maf^EX^ neurons (hM3Dq: c-Maf^EX^: n = 8; control: n = 5; Table S1). Responses to mechanical (H, K, L), heat (I), and cold (J). (M) Quantification of aversive behavior in c-Maf^EX^ and control mice injected with rAAV. EF1α.C_on_/D_on_.hM3Dq, 2 h after CNO injection (c-Maf^EX^: n = 8; control: n = 8). Error bars denote ±SEM. Number of mice and statistics are shown in Table S1. In brief: ^∗^p < 0.05, ^∗∗^p < 0.01 (B–L: ANOVA, followed by pairwise comparisons; M: unpaired Student’s t test). Scale bars, 100 μm (overview image) and 10 μm (higher-magnification images).

To examine potential roles of c-Maf deep dorsal horn neurons in the processing of somatosensory stimuli, we examined the consequences of transient silencing of c-Maf^EX^ or c-Maf^IN^ neurons in a battery of sensory tests. To this end, we injected an rAAV encoding the Cre/Dre double-dependent inhibitory chemogenetic receptor hM4Di (rAAV.hSyn1.C_on_/D_on_hM4Di-mCherry)^36^^,^^37^ into the lumbar spinal cord of c-Maf^EX^, c-Maf^IN^, or control mice (lacking either one or both recombinases) (Figure 4A). To determine the specificity and efficacy of the C_on_/D_on_hM4Di-mCherry construct, we reacted spinal slices from c-Maf^EX^ and c-Maf^IN^ mice injected with the AAV.C_on_/D_on_hM4Di-mCherry with anti-mCherry, c-Maf ,and Pax2 antibodies. We found 75% ± 2.6% of the excitatory mCherry^+^ (mCherry^+^;Pax2^−^) and 77.4% ± 3.1% of the inhibitory mCherry^+^ (mCherry^+^;Pax2^+^) cells to contain detectable levels of c-Maf (Figure 4A). Vice versa, 54.5% ± 4.2% of the c-Maf^EX^ cells (c-Maf^+^, Pax2^−^) and 56.3% ± 3.3% of the c-Maf^IN^ cells expressed detectable levels of mCherry. None of the mCherry^+^ neurons detected after injection into c-Maf^EX^ mice co-expressed Pax2. Starting 14 days after the intraspinal injection, mice were treated intraperitoneally (i.p.) with the chemogenetic agonist clozapine N-oxide (CNO) followed by sensory testing. Silencing of c-Maf^EX^ neurons did not change the responses of the mice to innocuous (Figures 4B and 4F; Table S1) or noxious mechanical stimuli (Figure 4E and Table S1), or noxious heat (Figure 4C and Table S1) or cold (Figure 4D) stimuli, nor did it alter performance in the rotarod test (Figure 4G and Table S1). Thus, silencing c-Maf^EX^ interneurons did not alter somatosensory thresholds in naive mice and did not impair gross motor coordination. Silencing of c-Maf^IN^ neurons reduced sensory thresholds to punctate mechanical stimulation (Figure S6B, von Frey; Table S1) but had no impact on thresholds of noxious thermal (heat and cold) or noxious mechanical (pin prick) stimuli (Figures S6C–S6F and Table S1).

### Activation of c-Maf^EX^ and c-Maf^IN^ neurons in naive mice

We next investigated behavioral effects of activation of c-Maf^EX^ or c-Maf^IN^ interneurons. To this end, we used a Cre/Dre double-dependent rAAV encoding the excitatory chemogenetic receptor hM3Dq^36^^,^^37^ (rAAV.hsyn1.C_on_/D_on_hM3Dq) (Figure S6A). Transient activation of c-Maf^EX^ neurons led to a strong reduction in mechanical thresholds in the von Frey test (Figure 4H), but no differences were observed in response to pin prick, brush, or heat stimulation (Figures 4I, 4K, and 4L; Table S1). Animals displayed a slight hypersensitivity to cold (Figure 4J and Table S1). Consistent with the observed mechanical hypersensitivity, activation of c-Maf^EX^ neurons also led to profound spontaneous aversive behaviors, including biting and flinching of the ipsilateral hindpaw (Figure 4M and Table S1). After repeated CNO injections, c-Maf^EX^ mice also developed skin lesions (Figure S7), which together with the increased biting behavior may indicate the presence of itch-like sensations after chemogenetic activation of c-Maf^EX^ neurons. Taken together, our data suggest that c-Maf^EX^ neurons are dispensable for noxious stimulus-evoked responses in naive mice, but their chemogenetic activation induces strong mechanical hypersensitivity and spontaneous aversive behaviors. Activation of c-Maf^IN^ neurons (Figures S6G–S6K) conversely reduced responses to noxious mechanical and dynamic mechanical stimuli (Figures S6J and S6K; Table S1).

### Mechanical nociception in neuropathic mice requires dorsal horn c-Maf^EX^ neurons

**Figure 5:**
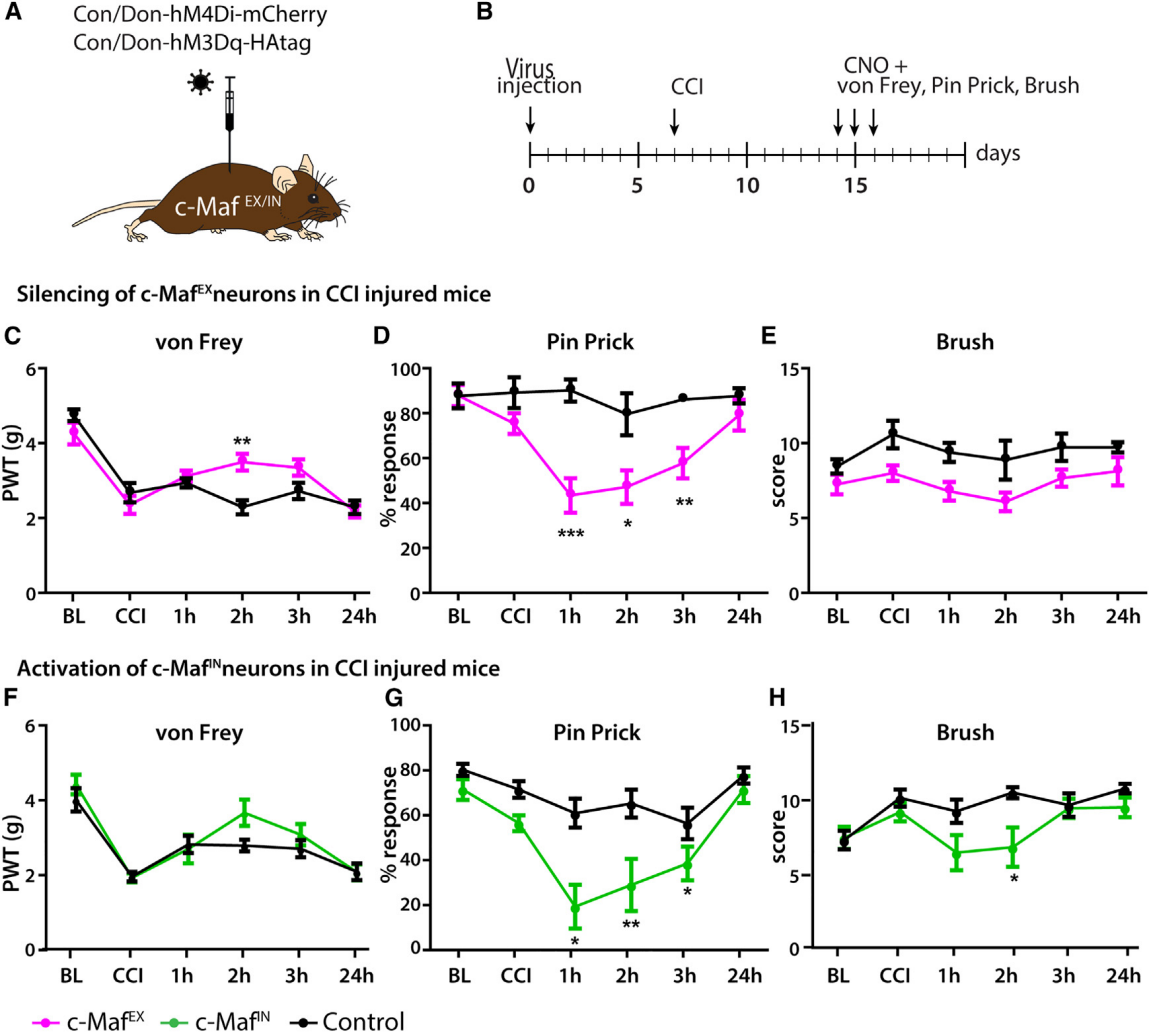
Pharmacogenetic modulation of c-Maf spinal interneuron activity in chronic pain states (A) DREADD expression was driven by injection of rAAV.EF1α.C_on_/D_on_.hM4Di (c-Maf^EX^ mice) or rAAV.EF1α.C_on_/D_on_.hM3Dq (c-Maf^IN^ mice) into the lumbar spinal cord of c-Maf^EX^, c-Maf^IN^, and control mice. (B) Virus injection was followed by CCI to induce neuropathic pain. (C–E) Responses to mechanical stimulation using the von Frey (C), pin prick (D), or light brush (E) tests before and after induction of neuropathic pain with CCI and silencing of c-Maf^EX^ neurons (c-Maf^EX^: n = 8; control: n = 7; Table S1). (F–H) Responses to mechanical stimulation using the von Frey (F), pin prick (G), or light brush (H) tests before and after induction of neuropathic pain with CCI and activation of c-Maf^IN^ neurons (c-Maf^IN^: n = 8; control: n = 8; Table S1). PWL, paw withdrawal latency; BL, baseline before injury; CCI, BL 7 days after chronic constriction injury and before CNO injection. 1 h to 24 h refers to time post CNO injection. Error bars denote ±SEM. Number of mice and statistics are shown in Table S1. In brief: ^∗^p < 0.05, ^∗∗^p < 0.01, ^∗∗∗^p < 0.001 (ANOVA, followed by pairwise comparisons).

The experiments described above suggest that c-Maf^EX^ neurons receive input from non-nociceptive touch-sensitive sensory fibers and connect to dorsal horn structures transmitting noxious mechanical stimuli but are silenced during acute nociceptive stimulation in naive mice. Previous work has suggested that under pathological conditions, such as after nerve injury and potentially also in response to peripheral inflammation, touch-sensitive sensory fibers gain access to dorsal horn nociceptive circuits giving rise to mechanical allodynia.^3^^,^^5^^,^^20^ We therefore asked whether c-Maf^EX^ neurons might be part of such allodynia circuits. To test this hypothesis, we examined the consequences of c-Maf^EX^ neuron silencing on nociception in neuropathic or inflammatory pain models. Neuropathic pain was induced in c-Maf^EX^ and control mice by a chronic constriction injury (CCI) of the left sciatic nerve.^39^ To inhibit c-Maf^EX^ neurons, animals were injected with the rAAV encoding the Cre/Dre double-dependent inhibitory hM4Di receptor (Figure 5A). CCI surgery was performed 1 week after injection of the rAAV (Figure 5B). Seven days after the CCI surgery, all mice displayed strong hypersensitivity to von Frey filament stimulation (Figure 5C and Table S1). Transient silencing of c-Maf^EX^ neurons significantly reduced this hypersensitivity (Figure 5C and Table S1) and, even more, the responses to pin prick stimulation (Figure 5D and Table S1). Responses to innocuous brush stimulation were not significantly affected (Figure 5E and Table S1).

Inflammatory hyperalgesia was evoked by subcutaneous plantar injection of zymosan A into the left hindpaw.^40^ Zymosan A was injected 10 days after the AAV injection, and mice were tested during the following 2 days (Figures S8A and S8B). Mice developed mechanical hypersensitivity within 24 h but, in contrast to what had been observed in mice after nerve injury, neither responses to von Frey filament stimulation nor responses to noxious pin prick stimulation were reduced by c-Maf^EX^ neuron silencing (Figures S8C–S8E and Table S1). Our data therefore indicate that c-Maf^EX^ neurons are elements of circuits of mechanical nociception and act as second-order mechano-nociceptors after nerve injury but do not contribute to mechanical sensitization induced by inflammation.

Silencing of c-Maf^IN^ neurons in naive mice produced mechanical hypersensitivity. We therefore investigated whether their activation after nerve injury would conversely reduce mechanical hypersensitivity in neuropathic mice. We indeed observed a profound reduction in pin-prick-evoked responses (Figure 5G and Table S1) and a slight, statistically non-significant reduction in mechanical (von Frey) hypersensitivity (Figure 5F and Table S1). These changes were strikingly similar to what was observed after the silencing of c-Maf^EX^ neurons.

### Inhibitory PV and c-Maf neurons control activity of c-Maf^EX^ neurons

**Figure 6:**
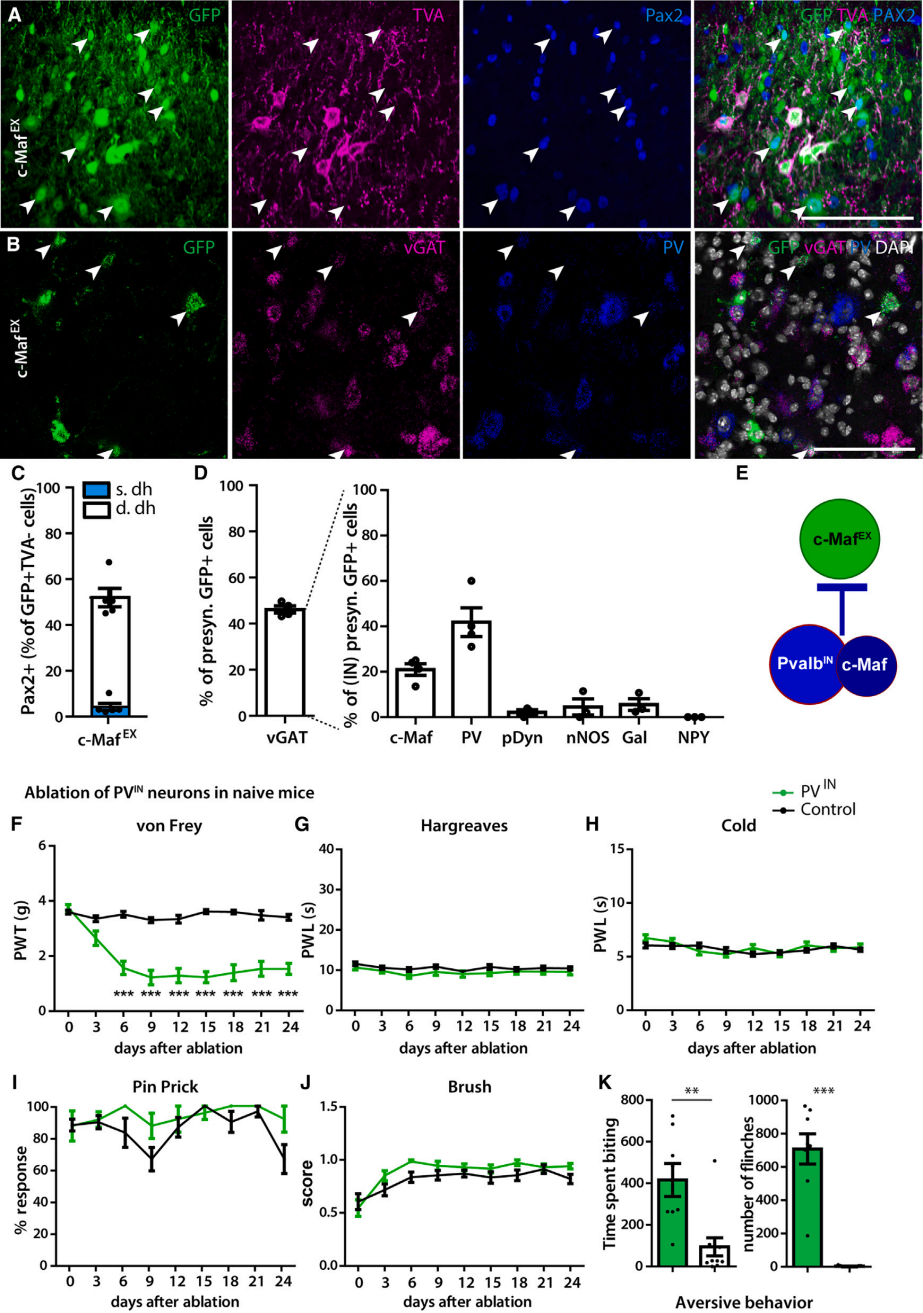
Inhibitory PV neurons control c-Maf^EX^ neurons and produce mechanical hypersensitivity, spontaneous pain, and itch after ablation (A) Immunofluorescence staining of spinal cord sections showing overlap between eGFP, TVA, and Pax2. (B) Triple *in situ* hybridization showing the overlap between eGFP, vGAT, and PV. (C) Quantification of the number of retrogradely labeled cells (eGFP^+^TVA^−^) that are inhibitory (Pax2^+^) in (A) (n = 5 mice, 1,086 neurons). (D) Quantification of (B) (vGAT: n = 4, 985 cells) and quantification of other spinal interneuron markers in the labeled inhibitory presynaptic neurons (see Figure S7) (c-Maf: n = 4, 326 cells; PV: n = 4, 289 cells; pDyn: n = 3, 603 cells; nNOS: n = 3, 194 cells; Gal: n = 3, 207 cells; NPY: n = 3, 220 cells). (E) Schematic illustration suggesting inhibitory PV and c-Maf neurons provide feedforward inhibition to c-Maf^EX^ neurons. (F–K) Behavioral responses after ablation of PV^IN^ neurons to mechanical (von Frey, F), heat (Hargreaves, G) or cold (dry ice, H), pin prick (I), and light brush (J) stimulation after ablation (in days) (PV^IN^: n = 12; control: n = 16). (K) Quantification of aversive behavior in PV^IN^ ablated and control mice at 12 days after DTX injection (PV^IN^: n = 8; control: n = 11). The time (s) spent liking/biting the injected paw and the number of flinches is quantified over 30 min. Arrowheads in (A) and (B): examples of Pax2^+^ neurons; thin arrows: examples of Pax2^−^ neurons. s.dh, superficial dorsal horn; IN, inhibitory. Error bars denote ±SEM. Number of mice and statistics are shown in Table S1. ^∗^p < 0.05, ^∗∗^p < 0.01, ^∗∗∗^p < 0.001 (F: ANOVA, followed by pairwise comparisons; K: unpaired Student’s t test). Scale bars, 100 μm.

The observation that chemogenetic activation of c-Maf^EX^ neurons produced allodynia and spontaneous pain-like behaviors in healthy mice, while their inhibition was without obvious effects, suggests that c-Maf^EX^ neurons are silenced under physiological conditions. To search for such inhibitory input, we used monosynaptic rabies tracing with c-Maf^EX^ neurons as the starter population. Primary infected neurons (c-Maf^EX^, eGFP^+^, TVA^+^ neurons) and neurons presynaptic to the starter population (eGFP^+^ but TVA^−^) could be distinguished by the respective presence or absence of TVA immunoreactivity (Figure 6A). About half of the eGFP^+^ TVA^−^ neurons were positive for Pax2 (52.3% ± 4.0%, Figures 6A and 6C). This was confirmed by *in situ* hybridization showing that 46.2% ± 1.5% of *eGFP*^+^ neurons also expressed *vGAT* (Figures 6B and 6D), suggesting a large inhibitory input onto c-Maf^EX^ neurons from local interneurons. We further investigated the identity of these inhibitory neurons using multiplex *in situ* hybridization (Figure 6D). We found that 21.0% ± 2.6% of the presynaptic inhibitory neurons also expressed *c-Maf* (Figure 6D) and 41.9% ± 6.3% expressed *PV* (Figures 6B and 6D). Other previously established markers for inhibitory dorsal horn interneurons such as *pDyn*, *nNOS*, *Gal*, and *NPY*^41^^,^^42^^,^^43^ were only expressed in few labeled neurons or in none at all (*pDyn*: 2.1% ± 1.1%; *nNOS*: 4.5% ± 3.5%; *Gal*: 5.6% ± 2.6%; *NPY*: 0.0% ± 0.0%, respectively; Figure 6D). c-Maf^EX^ neurons therefore appear to be predominantly controlled by inhibitory c-Maf and PV neurons (Figure 6E). These tracing results are in good agreement with the antihyperalgesic effect of c-Maf^IN^ neuron activation in neuropathic mice. Furthermore, the effect of c-Maf^EX^ neuron silencing was remarkably similar to that of c-Maf^IN^ neuron excitation, consistent with direct inhibition of c-Maf^EX^ by c-Maf^IN^ neurons.

However, silencing c-Maf^IN^ neurons in naive mice only partially recapitulated the behavioral effect of c-Maf^EX^ neuron activation suggesting additional inhibitory input to c-Maf^EX^ neurons, for example by inhibitory PV neurons. Consistent with this concept, a previous study reported the development of mechanical allodynia after ablation of all (excitatory and inhibitory) PV interneurons, yet no spontaneous pain or itch-like behavior was observed.^19^ We found that about 20% of all dorsal horn PV neurons are excitatory (Figures S9A–S9C) and that some c-Maf^EX^ neurons also express PV (Figure 1G). The remaining ∼80% of spinal dorsal PV neurons co-express GlyT2 (Figures S9B and S9C). We therefore decided to specifically ablate inhibitory PV (PV^IN^) neurons in the spinal cord, again using intersectional genetics. We generated PV^Dre^; GlyT2:Cre (PV^IN^ mice) and ablated PV^IN^ neurons by injecting AAV encoding a Cre/Dre double-dependent version of an inducible diphtheria toxin receptor (iDTR) cassette (AAV.hSyn.flex.roxed-iDTR) followed by i.p. injection of diphtheria toxin (DTX) 10 days later (Figure S9D). Seven to ten days after ablation, the number of PV neurons was reduced at the injected side (Figure S9E), resulting in a 42.3% loss of inhibitory interneurons in the deep dorsal horn (68.1 ± 8.3 vs. 118.2 ± 4.0 neurons per 25 μm spinal cord section, Figure S9F). Most of the remaining PV neurons were excitatory (Figure S9E). After PV^IN^ neuron ablation, mice showed a strong reduction in withdrawal thresholds upon von Frey filament stimulation (Figure 6F and Table S1) and developed strong spontaneous aversive behaviors such as biting and flinching of the affected hindpaw (Figure 6K and Table S1). However, responses to heat, cold, pin prick, and brush stimulation remained unchanged (Figures 6G–6J and Table S1). This phenotype hence recapitulated the behavioral changes observed after activation of c-Maf^EX^ neurons.

Taken together, we have shown that c-Maf^EX^ neurons receive most of their inhibitory input from local PV and c-Maf interneurons. Inhibiting either of these inhibitory populations produced phenotypes consistent with the idea that they gate c-Maf^EX^ neuron activity and thus prevent c-Maf^EX^ neurons from engaging nociceptive circuits in naive mice (Figure 6E).

### Output of c-Maf^EX^ neurons

**Figure 7:**
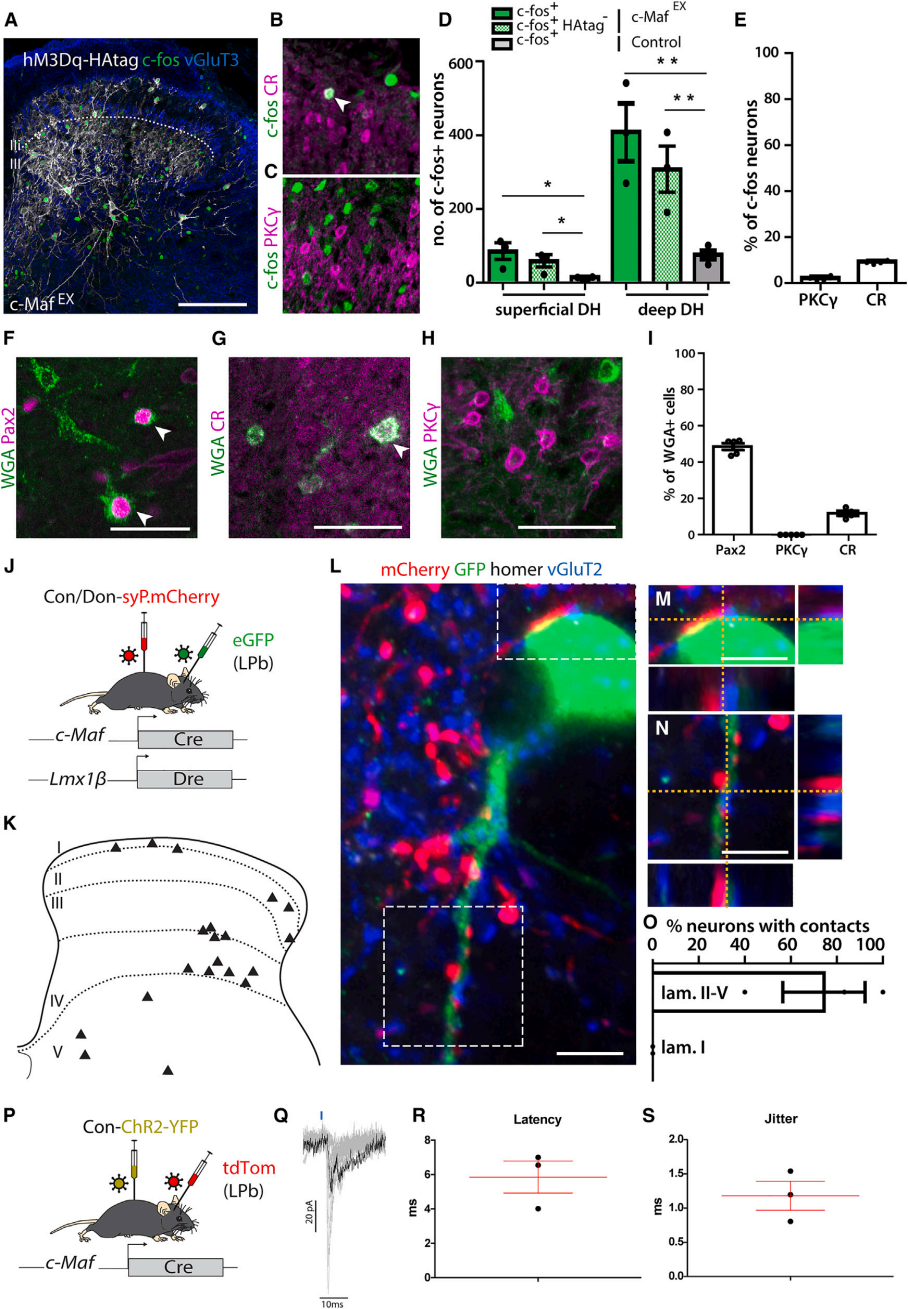
Anterograde tracing and labeling of the output of c-Maf^EX^ neurons (A) c-fos immunoreactivity in the spinal cord after injection of CNO in hM3Dq-expressing c-Maf^EX^ mice. Scale bars, 100 μm. (B) Immunohistochemistry showing overlap (white arrowhead) between c-fos^+^ and CR neurons after hM3Dq-mediated activation of c-Maf^EX^ neurons. (C) Immunohistochemistry showing no overlap between c-fos^+^ and PKCγ neurons. (D) Quantification of the number of c-fos^+^ neurons after activation of c-Maf^EX^ neurons (A) or control mice (c-Maf^EX^: n = 3, 1,219 deep and 245 superficial laminae neurons; control: n = 4, 293 deep and 46 superficial laminae neurons). (E) Quantification of the proportion of c-fos^+^ neurons expressing PKCγ or CR (n = 4, 3,175 and 2,458 c-fos^+^ neurons, respectively). (F–I) WGA-based anterograde tracing from c-Maf^EX^ neurons: *c-MafEX*; Rosa26^dstdTom/wt^ mice were injected with a virus carrying a Cre- and Dre-dependent WGA transgene. (F–H) Immunofluorescence staining on transversal sections of lumbar spinal cord, showing overlap between WGA^+^tdTom^−^ (postsynaptic to primarily infected *c-MafEX* neurons) neurons with Pax2 (F, white arrowheads), CR (G, white arrowhead), and PKCγ (H). Scale bars, 50 μm. (I) Quantification of (F)–(H) (n = 5, PKCγ: 834 cells; Pax2: 1,316 cells; CR: 1,054 cells). (J–O) Output of c-Maf^EX^ neurons onto spinoparabrachial projection neurons: synaptic terminals of c-Maf^EX^ neurons were labeled by intraspinal injection of ssAAV.hEF1α.-D_on_/C_on_.mSyp1_mCherry, and spinoparabrachial neurons by LPb injection of rAAV-retro/2-CAG-eGFP (J). (K) Diagram showing the localization of eGFP^+^ spinoparabrachial neurons labeled after injection. (L) Co-labeling of an eGFP^+^ neuron and mCherry^+^VGlut2^+^ synaptic terminals with the postsynaptic marker homer in the spinal cord. Scale bars, 5 μm. (M and N) Insets from (L) showing close apposition between mCherry^+^VGlut2^+^ and an eGFP^+^ spinoparabrachial neuron. Scale bars, 5 μm. (O) Quantification of the number of eGFP^+^ neurons receiving direct contacts from mCherry^+^ synaptic terminals (n = 3 mice; 19 eGFP^+^ neurons). (P–S) Electrophysiological recordings of optically induced excitatory postsynaptic currents (EPSCs) from retrogradely labeled LPb-projecting neurons in the spinal dorsal horn. (P) Schematic illustration of the injection strategy to express ChR2-YFP in c-Maf neurons and label LPb neurons. (Q) Example traces recorded in deep dorsal horn projection neurons after optogenetic stimulation in lumbar spinal cord sections. (R and S) Quantification of EPSC latencies and jitter after optogenetic stimulation (n = 3). Error bars denote ±SEM.^∗^p < 0.05, ^∗∗^p < 0.01 (unpaired Student’s t test). Scale bars, 100 μm.

Our data suggest that c-Maf^EX^ neurons act as second-order mechano-nociceptors after forced chemogenetic activation or after release from local inhibition. We next addressed the nature of the output of c-Maf^EX^ neurons to determine whether their target neurons are known components of circuits transmitting noxious information. To this end, we used orthogonal approaches. We quantified c-fos induction after chemogenetic activation of c-Maf^EX^ neurons and performed anterograde tracing employing wheat germ agglutinin (WGA) expression.^44^ Chemogenetic activation of c-Maf^EX^ neurons strongly increased the number of c-fos immunoreactive cells in the lumbar dorsal spinal cord (55.0 ± 16.6 vs. 11.5 ± 1.8 c-fos^+^ cells in the superficial laminae and 305.0 ± 62.8 vs. 73.2 ± 10.7 c-fos^+^ cells in the deep dorsal horn, Figures 7A–7D). Chemogenetically stimulated c-Maf^EX^ neurons thus provide excitatory input to nearby deep dorsal horn neurons and also relay excitation from the deep to the superficial dorsal horn. To further characterize these downstream neurons, we examined the expression of CR and PKCγ, marker genes of known separate excitatory interneuron populations that link deep dorsal horn neurons to the more superficially located nociceptive circuits.^12^^,^^13^^,^^45^ Only 1.8% ± 0.6% expressed PKCγ, but 8.9% ± 0.4% of c-fos^+^ neurons were positive for CR.

For anterograde tracing experiments with WGA, we injected a rAAV encoding a Cre/Dre double-dependent WGA transgene into the spinal cord of c-Maf^EX^ mice crossed to the Cre and Dre double-dependent reporter line Ai66 (Rosa26^dstdTom/wt^). The tdTomato reporter line was used to distinguish trans-synaptically labeled (tdTom^−^) from directly infected neurons (tdTom^+^). We found that about half of the targeted neurons (trans-synaptically labeled) expressed Pax2 (48.6% ± 1.8% of postsynaptic WGA^+^, Figures 7F and 7I). CR was found in 11.8% ± 1.4% of postsynaptic WGA^+^ neurons (Figures 7G and 7I). In contrast, none of the postsynaptic WGA^+^ neurons (tdTomato^−^) expressed PKCγ (Figures 7H and 7I). This is in line with our observation that some CR^+^, but almost no PKCγ^+^, neurons, were activated (c-fos^+^) following activation of c-Maf^EX^ neurons (Figure 7E).

Finally, we asked whether c-Maf^EX^ neurons could directly target ascending projection neurons of the dorsal horn. To this end, we labeled the synaptic terminals of c-Maf^EX^ neurons with the fusion protein synaptophysin-mCherry while spinoparabrachial neurons (and potentially fibers of passage^46^) were labeled from the (contra-)lateral parabrachial nucleus (LPb) using a rAAV2-retro-CAG-eGFP, a serotype that was specifically developed for improved axon terminal infection and retrograde transduction^47^ (Figure 7J). Many of the eGFP-labeled neurons were in the deep dorsal horn and a few scattered neurons were found in lamina I (Figure 7K). Of all eGFP^+^ LPb projection neurons in the deep dorsal horn, 62.7% ± 15.1% had at least one mCherry^+^VGlut2^+^ terminal in close apposition to a homer^+^ puncta on the cell body or a dendrite (Figures 7L–7O). No direct input of c-Maf^EX^ neurons onto lamina I projection neurons was detected. To demonstrate the functionality of the connections between c-Maf^EX^ neurons and ascending deep dorsal horn projection neurons, we overexpressed ChR2-YFP (AAV.Flex.ChR2-YFP) in spinal c-Maf neurons (Figure 7P) and recorded light-evoked potentials in spinal projection neurons retrogradely labeled from the LPb with AAV2retro.tdTomato. Recorded cells responded with an average latency of 5.9 ± 0.9 ms (jitter: 1.2 ± 0.2 ms), suggesting monosynaptic connections between c-Maf^EX^ neurons and deep dorsal horn neurons projecting to the LPb (Figures 7O–7S). Taken together, our results suggest that c-Maf^EX^ neurons can engage nociceptive circuits via two pathways: activation of CR neurons in superficial laminae and direct activation of scattered deep dorsal LPb projection neurons.

## Discussion

The present study has focused on the function of dorsal horn c-Maf neurons in spinal nociceptive signaling. These neurons are of particular interest, as they constitute a main target population of the corticospinal tract whose activity is required for nerve-injury-induced neuropathic mechanical sensitization.^8^ We demonstrate that c-Maf neurons fall into two subpopulations, excitatory and inhibitory interneurons, which exert opposing effects on pain. c-Maf^EX^ neurons are part of a normally silent circuit required for nerve-injury-induced mechanical allodynia, and relays signals from non-nociceptive sensory fibers to dorsal horn nociceptive output structures. Under healthy conditions, these c-Maf^EX^ neurons are silenced via feedforward inhibition by c-Maf^IN^ and inhibitory PV interneurons.

Disinhibition as a source for functionalization of c-Maf^EX^ neurons is in good agreement with previously proposed mechanisms for neuropathic sensitization.^48^^,^^49^ However, disinhibition also occurs in inflammation.^50^^,^^51^ Yet silencing of c-Maf^EX^ neurons failed to reduce allodynia in mice with inflamed paws, supporting the concept that distinct allodynia circuits become activated depending on the nature of the underlying pathology.^10^

### Integration of c-Maf^EX^ and c-Maf^IN^ neurons in sensory circuits of the dorsal horn

c-Maf^EX^ neurons receive input from myelinated primary afferent fibers including TrkB-positive LTMRs. Input from these fibers has previously been shown to be essential for nerve-injury-induced mechanical allodynia.^52^ The prevalence of this non-nociceptive input to c-Maf neurons is consistent with the location of c-Maf neurons in the deep dorsal horn. Analyses of changes in c-fos expression following chemogenetic c-Maf^EX^ neuron activation and anterograde tracing with WGA revealed that CR neurons but not PKCγ neurons, another subpopulation previously reported to affect nerve-injury-evoked pain sensitivity,^53^ are postsynaptic to c-Maf^EX^ neurons. CR neurons have previously been proposed to connect VGlut3 lineage neurons to nociceptive circuits of the superficial dorsal horn.^11^ CR neurons may therefore act as third-order interneurons in a pathway that relays LTMR input to dorsal horn nociceptive output neurons via c-Maf^EX^ neurons. This is consistent with recent studies indicating that CR neurons receive polysynaptic input from Aβ fibers (LTMRs) and in turn directly target spinoparabrachial projection neurons in lamina I.^12^^,^^45^^,^^54^

In addition to the superficial dorsal horn output system, our study identified a second output pathway that links c-Maf^EX^ neurons to higher-order nociceptive centers. c-Maf^EX^ neurons frequently contacted scattered deep dorsal horn projection neurons that were retrogradely labeled from the LPb. While most recent work has focused on lamina I projection neurons, projection neurons in the deep dorsal horn act as an additional nociceptive output system.^55^ In addition, work conducted by Browne et al.^56^ suggests that laminae (III–V) projection neurons form an alternative route to provide qualitatively different sensory information to the LPb. Our data therefore suggest that excitatory deep dorsal horn c-Maf interneurons may engage pain circuits by directly activating deep dorsal horn projection neurons, independently of the superficial dorsal horn.

Our finding that ablation or silencing of c-Maf^EX^ neurons had no major impact on nociceptive behavior in healthy mice suggests the presence of strong inhibitory control of these neurons. Retrograde tracing experiments performed to reveal the origin of such inhibition identified inhibitory PV and c-Maf^IN^ neurons presynaptic to c-Maf^EX^ neurons. Compromising the activity of these neurons led to mechanical hypersensitivity and spontaneous aversive behaviors reminiscent of those evoked by the activation of c-Maf^EX^ neurons. Given that the sensory input to c-Maf^EX^ and c-Maf^IN^ neurons is very similar, we propose that c-Maf^IN^ neurons provide feedforward inhibition to c-Maf^EX^ neurons. The results of our c-Maf^IN^ and PV^IN^ neuron inhibition and ablation experiments are consistent with a previous study indicating that loss of spinal PV neurons leads to mechanical hypersensitivity.^19^ However, in this study, no spontaneous pain-like or itch-like behavior was reported. This might be because in this previous study all PV neurons were ablated, which included about 15% excitatory neurons that may be necessary for the observed spontaneous aversive behaviors.

Our retrograde tracing experiments revealed additional sources of input to dorsal horn c-Maf^EX^ neurons originating from supraspinal centers, including the primary somatosensory cortex S1, the red nucleus, and the RVM. In the context of the present study, the primary somatosensory cortex S1 is of particular interest because transection of the corticospinal tract specifically abolishes mechanical hyperalgesia in neuropathic mice.^8^ This specificity fits with the innervation of dorsal horn c-Maf^EX^ neurons by low-threshold mechanoreceptors and the virtual absence of input from unmyelinated nociceptors.

Our data indicate that c-Maf^EX^ and c-Maf^IN^ neurons are innervated by a similar set of peripheral as well as supraspinal neurons. This leads us to propose that c-Maf^IN^ neurons provide feedforward inhibition to c-Maf^EX^ neurons. However, this raises the conundrum of how a disynaptic connection can inhibit monosynaptic input from the same source. We provided evidence that the optogenetic innervation of CST terminals produced light-evoked responses in c-Maf^EX^ neurons with almost twice the latency as compared with c-Maf^IN^ neurons. Differential dendritic filtering of peripheral and supraspinal input to c-Maf^EX^ and c-Maf^IN^ neurons as suggested by Zhang et al.^57^ might result in effective feedforward inhibition. Clearly, additional studies are required to address interconnectivity, for example between supraspinal sites and spinal neurons, in greater detail.

### c-Maf neurons and neuropathic itch

c-Maf^EX^ neuron activation experiments revealed phenotypes that were reminiscent of neuropathic itch in human patients. In humans, nerve injury not only gives rise to neuropathic hyperalgesia and allodynia but can also lead to neuropathic itch (allokinesis).^58^ It has been suggested that similar mechanisms underlie both pathologies. This concept is backed by our finding that activation of c-Maf^EX^ neurons produces both neuropathic pain-like behaviors (i.e., spontaneous pain and mechanical allodynia) and itch-like behaviors (biting leading to self-inflicted skin lesions). Furthermore, both phenotypes are recapitulated by the ablation of inhibitory PV neurons. Together with our retrograde tracing experiments, these data suggest that, under disinhibitory conditions, LTMR input can give rise to pain-like or itch-like behavior, both depending on the recruitment of dorsal horn c-Maf^EX^ neurons.

In summary, our study has identified c-Maf^EX^ neurons as critical elements of a spinal allodynia and allokinesis circuit that connects innocuous input from touch-sensitive sensory fibers to dorsal horn nociceptive output structures. c-Maf^EX^ neurons appear to serve a unique function in this circuit by integrating peripheral sensory input with both local inhibition and descending excitation from the corticospinal tract. They hence link this circuit not only to well-established disinhibitory processes in neuropathic pain but also to more recent concepts pointing to the importance of top-down modulation in neuropathic pain.^8^

### Limitations of the study

In this study, we demonstrate that deep dorsal horn c-Maf^EX^ neurons connect non-nociceptive somatosensory input to spinal nociceptive output pathways. We propose that the activity of these c-Maf^EX^ neurons is normally silenced by inhibitory PV and c-Maf^IN^ neurons. This model is based on retrograde tracing experiments and the correlation of c-Maf^EX^ gain-of-function and PV^IN^ and c-Maf^IN^ loss-of-function experiments. To ultimately prove that loss of PV^IN^ and c-Maf^IN^ function leads to c-Maf^EX^ activation, combinatorial gain- and loss-of-function experiments would be required. However, this requires the identification of non-overlapping marker genes for the respective populations. We also demonstrate that c-Maf^EX^ neurons are critical for mechanical hypersensitivity and allodynia after nerve injury. Compared with silencing other excitatory dorsal horn populations, e.g., CCK^+^ or VGlut3^+^ neurons,^8^^,^^10^ silencing c-Maf^EX^ neurons had a relatively small impact on mechanical allodynia, although we and others reported an extensive overlap between c-Maf^EX^ and CCK^+^ or VGlut3^+^ neurons.^10^ Methodological differences (chemogenetic silencing vs. toxin-mediated ablation or silencing) may account for these differences.

## STAR★Methods

### Key resources table

**Table:** 

REAGENT or RESOURCE	SOURCE	IDENTIFIER
**Antibodies**		

rabbit anti-GFP (1:1000)	Molecular Probes	AB_221570; RRID:AB_221570
rabbit anti-c-Maf (1:1000)	Dr Carmen Birchmeier	Wende et al.^23^
rabbit anti-PV (1:1000)	Immunostar	AB_572259; RRID:AB_572259
rabbit anti-NF200 (1:1000)	Sigma-Aldrich	AB_477272; RRID:AB_477272
rabbit anti-PKCγ (1:1000)	Santa Cruz	AB_632234; RRID:AB_632234
rabbit anti-CGRP (1:1000)	Immunostar	AB_572217; RRID:AB_572217
rabbit anti-c-fos (1:1000)	Santa Cruz	AB_2106765; RRID:AB_2106765
rabbit anti-WGA (1:50 000)	Sigma Aldrich	AB_261669; RRID:AB_261669
rabbit anti-TVA (1:1000)	Dr Sauer	Seidler et al.^59^
rabbit anti-homer (1:2000)	Synaptic Systems	AB_2120990; RRID:AB_2120990
rabbit anti-P2X3 (1:1000)	Abcam	AB_297006; RRID:AB_297006
rabbit anti-calretinin (1:1000)	Sigma-Aldrich	AB_2068506; RRID:AB_2068506
goat anti-TrkA (1:200)	R&D Systems (Minneapolis, MN, USA)	AB_2283049; RRID:AB_2283049
goat anti-TrkC (1:400)	R&D Systems (Minneapolis, MN, USA)	AB_2155412; RRID:AB_2155412
goat anti-Pax2 (1:400)	R&D Systems (Minneapolis, MN, USA)	AB_10889828; RRID:AB_10889828
goat anti-tdTomato (1:1000)	Sicgen	AB_8181-200; RRID:AB_2722750
guinea pig anti-Lmx1b (1:10 000)	Dr Carmen Birchmeier	Muller et al.^26^
guinea pig anti-NeuN (1:1000)	Synaptic Systems	AB_2619988; RRID:AB_2619988
guinea-pig anti-c-Maf (1:2000)	Dr Carmen Birchmeier	Wende et al.^23^
guinea pig anti- VGlut1 (1:2000)	Millipore	AB5905; RRID:AB_2301751
guinea pig anti- VGlut2 (1:1000)	Synaptic Systems	135404; RRID:AB_887884
guinea pig anti- VGlut3 (1:500)	Synaptic Systems	AB_2619825; RRID:AB_2619825
chicken anti-GFP (1:1000)	LifeTechnologies	AB_2534023; RRID:AB_2534023
Rat anti-mCherry (1:1000)	Abcam	AB_11133266; RRID:AB_11133266
Sheep anti-PlxnC1 (1:200)	R&D Systems (Minneapolis, USA)	AB_2284038; RRID:AB_2284038
isolectin IB4 (1:500)	Invitrogen Molecular Probes	AB_2314662; RRID:AB_2314662

**Bacterial and virus strains**		

AAV-9/2-hEF1α-C_on_/D_on_-eGFP	VVF (Zurich, Switzerland)	vHW18-9 Frezel et al.^14^
AAV-9/2-hEF1α-C_on_/D_on_-hM3Dq-HAtag	VVF (Zurich, Switzerland)	vHW24-9 (this publication)
AAV-8/2-hEF1α-C_on_/D_on_ -hM4Di-mCherry	VVF (Zurich, Switzerland)	vHW23-8 (this publication)
AAV-retro/2-CAG-EGFP	VVF (Zurich, Switzerland)	v24-retro
AAV-8/2-hEF1α/hTLV1- C_on_/D_on_-mSyp1-mCherry	VVF (Zurich, Switzerland)	vHW51-8 (this publication)
AAV9-CAG-ChR2-eYFP	VVF (Zurich, Switzerland)	v649
AAV9-EF1a-flex-ChrR2-YFP	VVF (Zurich, Switzerland)	v214
AAV-retro/2-hSyn-tdTomato	VVF (Zurich, Switzerland)	v272-retro
AAV-8/2-hSyn1-roxSTOP-dlox-TVA_2A.RabG	VVF (Zurich, Switzerland)	vHW18-9 Frezel et al.^14^
SAD.RabiesΔG.eGFP (EnvA) (EnvA.RV.dG.eGFP)	Salk Institute (La Jolla, CA, USA)	Albisetti et al.^60^
AAV1.EF1α-flex-rox.iDTR(HB-EGF).hGH	Penn Vector Core (Philadelphia, USA)	Lot V4555MI-S (this publication)
AAV-1/2-hSyn1-roxSTOP-dlox-WGA	IPT (Zurich, Switzerland)	vHW8-1 (this publication)

**Chemicals**, **peptides**, **and recombinant proteins**		

Clozapine-N-Oxyde (CNO)	Enzo Life Sciences (Farmingdale, NY, USA)	34233-69-7
DTX	Millipore (Burlington, MA, USA)	322326

**Critical commercial assays**		

RNAscope Fluorescent Multiplex Reagent Kit	Advanced Cell Diagnostics (ACD)	Cat No. 320850
c-Maf	ACD	412959-C2
CCK	ACD	402271-C1
CRE	ACD	312281-C3
GFP	ACD	400281-C1
RORα	ACD	520031-C2
VGlut2	ACD	319171-C1
VIAAT (vGAT)	ACD	319191-C1
VIAAT (vGAT)	ACD	319191-C2
GlyT2	ACD	409741-C1
PV	ACD	421931-C3
Calb2 (calretnin)	ACD	313641-C2
Trkb (Ntrk2)	ACD	423611-C3
Mrgpra3-O1	ACD	502041-C2
Gal	ACD	400961-C2
Pdyn	ACD	318771-C3
NPY	ACD	313321-C2
nNOS		437651-C2
Triplex positive control probe	ACD	3-plex Positive Control Probe- Mm
Triplex negative control probe	ACD	3-plex Negative Control Probe- Mm

**Deposited data**		

Raw data	This paper	Zenodo https://doi.org/10.5281/zenodo.7648723

**Experimental models**: **Organisms/strains**		

C57BL/6J (wild type)	The Jackson Laboratory	IMSR_JAX:000664
B6; 129S-Gt(ROSA)26Sortm66.1(CAG-tdTomato)Hze/J (Rosa26^dstdTom/wt^)	The Jackson Laboratory	IMSR_JAX:007914
Gt(ROSA)26Sortm2(CAG-NuTRAP)Evdr/J	The Jackson Laboratory	IMSR_JAX:029899
Pvalbtm3.1(dreo)Hze (Pvalb^Dre^)	The Jackson Laboratory	IMSR_JAX:021190
c-Maf^Cre/wt^	Dr Carmen Birchmeier	(this publication)
Glyt2::Cre	IPT (Zurich, Switzerland)	Foster et al.,^25^
Glyt2::Dre	IPT (Zurich, Switzerland)	Albisetti et al.^24^
Lmx1β^Dre^	IPT (Zurich, Switzerland)	(this publication)

IPT: Institute of Pharmacology and Toxicology, University of Zurich; VVF: Viral Vector Facility (University of Zurich; www.vvf.uzh.ch).

### Resource availability

#### Lead contact

Further information and requests for resources and reagents should be directed to and will be fulfilled upon reasonable request by Hendrik Wildner (hwildner@pharma.uzh.ch).

#### Materials availability

The transgenic mouse lines Lmx1b^Dre^and c-Maf^Cre^ are available upon reasonable request after signing a material transfer agreement with the University of Zurich (Lmx1b^Dre^) or the Max-Delbrück Center, Berlin (c-Maf^Cre^). AAVs generated in this study are available from Viral Vector Facility (University of Zurich; www.vvf.uzh.ch).

### Experimental model and subject details

Experiments were performed on 6-12-week-old mice kept at a 12:12 h light/dark cycle with ad libitum access to food and water. Permissions for experiments have been obtained from the Canton of Zurich (permissions 03/2018, 031/2016, and 063/2016).

#### Mouse lines

The c-Maf-Cre allele was generated by homologous recombination in embryonic stem (ES) cells as described.^61^ The c-*Maf* coding region was replaced by a cassette containing the *Cre* recombinase open reading frame followed by an FRT flanked neomycin resistance cassette (Figure 1C). Mutant ES cells were injected into blastocysts to generate the mutant mouse strains. The neomycin cassette was removed by crossing F1 mice with FLPe deleter mice.^62^ Homologous recombination and removal of the neomycin resistance cassette were verified by Southern blot analysis.

The Lmx1b^Dre^ mouse was generated using CRISPR-Cas9 gene targeting directly in mouse embryos. C57BL/6J female mice underwent ovulation induction by i.p. injection of 5 IU equine chorionic gonadotrophin (PMSG; Folligon–InterVet), followed by i.p. injection of 5 IU human chorionic gonadotropin (Pregnyl–Essex Chemie) 48 h later. For the recovery of zygotes, C57BL/6J females were mated with males of the same strain immediately after the administration of human chorionic gonadotropin. All zygotes were collected from oviducts 24 h after the human chorionic gonadotropin injection and were then freed from any remaining cumulus cells by a 1–2 min treatment of 0.1% hyaluronidase (Sigma-Aldrich) dissolved in M2 medium (Sigma-Aldrich). Mouse embryos were cultured in M16 medium (Sigma-Aldrich) at 37°C and 5% CO_2_. For micromanipulation, embryos were transferred into M2 medium. All microinjections were performed using a microinjection system comprised of an inverted microscope equipped with Nomarski optics (Nikon), a set of micromanipulators (Narashige), and a FemtoJet microinjection unit (Eppendorf). Injection solution containing sgRNA (300 ng/μL), Cas9 protein (IDT, 50 ng/μL) and the Lmx1b-P2A-Dre homologous recombination template plasmid (10 ng/μL) was microinjected into the male pronuclei of fertilized mouse oocytes until 20–30% distension of the organelle was observed. Embryos that survived the microinjection were transferred on the same day into the oviducts of 8–16-wk-old pseudopregnant Crl:CD1 (ICR) females (0.5 d after coitus) that had been mated with sterile genetically vasectomized males^63^ the day before embryo transfer. Pregnant females were allowed to deliver and raise their pups until weaning age.

GlyT2:Dre mice were generated using the same strategy as for the GlyT2:eGFP and GlyT2:Cre mouse lines.^24^^,^^25^^,^^64^ For further details on the genetically modified mice used in this study, see KRT.

### Method details

#### Immunohistochemistry (IHC)

Mice were transcardially perfused with 4% ice-cold paraformaldehyde (in 0.1M sodium phosphate buffer, pH 7.4). Lumbar spinal cords, brains and dorsal root ganglia (DRGs) were immediately dissected and post-fixed for 2.5 h with 4% paraformaldehyde (PFA) on ice. Post-fixed tissue was briefly washed with 0.1M sodium phosphate buffer (pH 7.4) and then incubated in 30% sucrose (in PBS) overnight at 4°C for cryoprotection. Cryoprotected tissue was cut at 25 μm, 40 μm or 16 μm (spinal cord, brain, or DRGs respectively) on a Hyrax C60 Cryostat (Zeiss, Oberkochen, Germany), mounted on superfrost plus glass microscope slides and then incubated with the respective combinations of primary antibodies in 1% donkey serum in phosphate buffered saline (PBS) overnight at 4°C. After brief washes in PBS, sections were incubated with the respective secondary antibodies for 2 h at room temperature and briefly rinsed in PBS, before mounting with coverslips and DAKO fluorescent mounting media (Dako, Carpinteria, CA, USA). Secondary antibodies raised in donkey were purchased from Jackson Immuno Research (West Grove, PA, USA). All primary antibodies used are listed in the KRT.

#### Multiplex *in situ* hybridization (ISH) and image analysis

Spinal cord and DRG tissues used for ISH were rapidly dissected from 6 - 12-week-old mice, collected in 1.5 mL Eppendorf tubes, and immediately frozen in liquid nitrogen. Tissues were embedded in NEG50 frozen section medium (Richard-Allen Scientific), cut into 16 μm sections, and hybridized using the probes designed for RNAscope Fluorescent Multiplex ISH listed in KRT.

For IHC and ISH analysis, image stacks of fluorescent images were acquired on a Zeiss LSM700 confocal and a Zeiss LSM800 Airy Scan microscope (Zeiss, Oberkochen, Germany). The number of immunoreactive cells in image stacks were determined using the ImageJ (NIH, Bethesda, Maryland) Cell Counter plugin (Kurt DeVos, University of Sheffield, Academic Neurology).

#### Slice preparation and electrophysiological recordings

Sagittal (300 μm) or transverse (400 μm) spinal cord slices were prepared using 6 - 8 week-old mice of both sexes. Spinal cords were extracted while the tissue was kept in an ice-cold solution containing (in mM): 65 NaCl, 105 Sucrose, 1.25 NaH2PO4, 25 NaHCO3, 2.5 KCl, 25 Glucose, 7 MgCl2, 0.5 CaCl2. Oxygen was provided to the fresh tissue by bubbling the solution with a mixture of 95% O_2_ and 5%CO_2_. Extracted spinal cords were then glued to an AGAR surface and subsequently sliced using a vibrating blade microtome (D.S.K., microslicer DTK 1000), the cutting direction was chosen according to the axis of preference (sagittal or transverse). Slices were then transferred and maintained in 37°C artificial cerebrospinal fluid (aCSF) containing (in mM): 120 NaCl, 2.5 KCl, 1.25 NaH_2_PO_4_, 26 NaHCO_3_, 5 HEPES, 1 MgCl_2_, 2 CaCl_2_ and 14.6 glucose (pH 7.4), equilibrated with 95% O_2_, 5% CO_2_.

Targeted whole-cell patch-clamp recordings from c-Maf^cre^; Lmx1b^Dre^/GlyT2:Dre neurons were performed at room temperature. Slices were superfused continuously with oxygenated aCSF throughout the duration of the recording at the rate of 1-2 mL/min.

For the biophysical characterization of c-Maf^cre^;Lmx1b^Dre^/GlyT2:Dre neurons, cells were identified through expression of tdTomato in c-Maf^cre^; Lmx1b^Dre^/GlyT2:Dre; Ai66 mice. Patch pipettes (borosilicate glass; 4–8 MΩ; Harvard Apparatus) were filled with intracellular solution containing (in mM): 130 K^+^-gluconate, 5 NaCl, 1 EGTA, 10 HEPES, 5 Mg-ATP, 0.5 Na-GTP (pH 7.35, 280–290 mosm/l). Passive and active properties were recorded in current-clamp mode. The value of resting membrane potential (RMP) was recorded immediately after switching from voltage-clamp to current-clamp. Capacitance was determined with repeated hyperpolarizing steps (−10 mV, 100 ms), recording transient capacitive current. From the current was then calculated the charge transfer, which was used to calculate the value of the capacitance using the capacitance-charge-voltage relation equation: capacitance (C) = charge (Q)/volts (V). Input resistance (R_input_) was determined through injection of hyperpolarizing steps (2 s, −5 pA increment, delivered every 10 s). The average of voltage values of the final 500ms of the hyperpolarizing response were plotted against the current steps values: the slope of the line fitting the plotting was identified as the R_input_. Action potential (AP) threshold was calculated at rheobase using dV/dt transformation. The value of the threshold was identified as the point where the increase in voltage from baseline was at least 5 mV/ms. After-hyperpolarization (AHP) was identified as the difference in voltage between the lowest point of the hyperpolarization phase and baseline, while AP width was measured as the time window between threshold and AHP peak.

AP firing patterns evoked by depolarizing current injection were classified according to previously published criteria.^65^ In brief, delayed (D) firing neurons were characterized by a delay between the onset of the depolarizing step and the AP discharge. Tonic (T) neurons were characterized by constant APs discharge lasting the whole duration of the depolarizing step. Neurons displaying a gap between series of AP discharges were classified as gap (G) firing and neurons with a burst of action potentials at the beginning of the depolarizing step as initial bursting (Ib) neurons.

In optogenetic experiments we activated ChR2 in acute slices by using an optic fiber (PlexBright optogenetic stimulation system patch cable. 200/230 μm fiber, Plexon, Inc.) directing blue light (470nm) toward the field of the slice that was recorded. The light was produced by a LED module (PlexBright LED module, 470 nm, Plexon, Inc) and the light intensity was controlled through a current generator (Plexon, LED Driver LD-1, Plexon, Inc) plugged to the LED module itself and controlled by the amplifier. The output current was set at the maximum value of 300mA, producing a light intensity of 2.5mW.

To study CST input onto c-Maf^cre^; Lmx1b^Dre^/GlyT2:Dre neurons we injected AAV.EF1a.ChR2-YFP into S1 of c-Maf^cre^; Lmx1b^Dre^/GlyT2:Dre, Ai66 mice and performed voltage-clamp experiments. For these we filled the patch pipettes with an internal solution containing (in mM): 120 CsCl, 10 HEPES, 10 EGTA, 4 MgCl_2_, 2 Mg-ATP, 0.5 Na-GTP, 5 QX-314 (pH 7.35, 280–290 mosm/l). The latency was determined between light onset and onset of the EPSC, while the jitter was calculated as the standard deviation of the latency values of twenty consecutive EPSCs. Light-evoked EPSCs were recorded at a holding potential of −70 mV. To study the input of excitatory c-Maf^cre^ neurons onto ascending projection neurons we injected the LPb of c-Maf^cre^ mice with an AAV2retro.tdTomato and the lumbar spinal cord with an AAV.EF1a.flex.ChR2-YFP. In subsequent voltage-clamp experiments an internal solution containing (in mM): 135 CsMethanesulfonate, 3 NaCl, 10 HEPES, 0.6 EGTA, 4 MgATP, 0.3 NaGTP, 5 QX-314 (pH 7.35, 280–290 mosm/l) was used. We isolated light evoked EPSCs in ascending projection neurons by clamping at a holding potential of −70 mV, where the chloride driving force is near to 0. All internal solutions contained 0.2% Biocytin. Data were acquired using an EPC9 amplifier (HEKA Elektronik, Lambrecht, Germany) controlled with Patchmaster, version 2x80 acquisition software and sampled at 20 kHz. Data were analyzed using IGOR Pro 6.22A.

#### AAV design and production

Cre and Dre dependent viral vectors were designed based on the INTRSECT approach.^38^^,^^66^ Viral particles were generated by the viral vector core facility Zurich (VVF).

#### Intraspinal and brain virus injections

Viruses were obtained from the resources indicated in the KRT and used as previously described.^67^ Virus injections were made in adult (6-8-week-old) mice anesthetized with 2% isoflurane and immobilized on a motorized stereotaxic frame (David Kopf Instruments, Tujunga, CA, USA and Neurostar, Tübingen, Germany). For intraspinal injections, the vertebral column was fixed using a pair of spinal adaptors and lumbar spinal cord at L4 and L5 was exposed. Injections (3 × 300 nL) spaced approximately 1mm apart were made at a rate of 50 nL/min through glass micropipettes (tip diameter 30–40 μm) attached to a 10 μL Hamilton syringe. For the parabrachial nucleus (LPb) injections, the head was fixed using ear bars, the skull exposed, and the following injection coordinates were used: (bregma −5.2 mm; midline +1.2 mm; depth: 3.4 mm).

#### Diphtheria toxin mediated ablation of PV^IN^ neurons

Double transgenic (PV^Dre^; GlyT2:Cre) and control mice were injected intraspinally with AAV1.EF1α.flex.roxed-iDTR(HB-EGF).hGH and received an i.p. injection of 50 μg/kg DTX suspension (in filtered 0.9% NaCl) 10 days after the intraspinal injection.

#### Behavioral analysis

Double transgenic male mice (c-Maf^Cre/wt^; Lmx1b^Dre/wt^ (c-Maf^EX^), c-Maf^Cre/wt^; GlyT2:Dre (c-Maf^IN^), and PV^Dre/wt^; GlyT2:Cre (PV^IN^), expressing both Cre and Dre) were compared to control (Cre^+^ only, Dre^+^ only or Cre^−^Dre^−^) mice, all injected with 2 mg/kg CNO. All behavioral tests were performed by an experimenter blinded to the genotype of the mice as previously described.^68^ Only one test was performed per day and mouse.

##### Mechanical sensitivity

Mice were placed in Plexiglas chambers (8 × 8 cm) on a raised wire grid and allowed to acclimatize for at least 1 h before testing. Withdrawal thresholds were assessed by the stimulation of the hind paw with an electronic von Frey anesthesiometer (IITC, Woodland Hills, CA). Eight measurements were taken at an interval of 10 min between stimulations. Sensitivity to light touch or acute painful stimulation was also tested. Both hind paws were stimulated alternately, and 10 measurements were taken from each hind paw. For light touch, mice were gently brushed with a soft paintbrush on the plantar surface of the hind paw. For acute painful stimulation, the plantar surface of hind paws was stimulated with a blunted G26 needle without penetration of the skin. For both tests, each response was given a score of 0 or 1 for no response or brief withdrawal of the paw and plotted as a percentage of positive responses (ie, a mouse that responded 8 out of 10 times gave a score of 80%).

##### Cold sensitivity

Mice were placed in Plexiglas chambers (8 × 8 cm) on a 5-mm thick borosilicate glass platform and allowed to acclimatize for at least 1 h before testing. A dry ice pellet was applied to the surface of the glass below the paw, cooling the surface. Withdrawal thresholds were measured using a stopwatch, and a cutoff time of 20 s was set.

##### Heat sensitivity (Hargreaves test)

Mice were placed in Plexiglas chambers (8 × 8 cm) on a glass surface and allowed to acclimatize for at least 1 h before testing. A movable infrared generator was positioned below the plantar surface of each hind paw alternately. Withdrawal thresholds were recorded with electronically controlled commercially available instrument with a built-in timer (Plantar Analgesia Meter; IITC, Woodland Hills, CA), and a cutoff time of 32 s was set. Eight measurements were taken at an interval of 10 min.

##### Spontaneous aversive behavior

Mice were placed in Plexiglas cylinders (Ø 15 cm × 24 cm). The number of flinches and the time spent licking were measured over a 30 min period.

##### Chronic pain models

Neuropathic pain was studied using the CCI model. Seven-to 8- week-old c-Maf^Ex^ mice and transgene negative control mice underwent constriction injury of the left sciatic nerve just proximal to the trifurcation was performed as described previously (Reference). Briefly, anesthesia was induced and maintained by 2% isoflurane (Provet AG, Lyssach, Switzerland), combined with oxygen (30%). Before the start of the surgery, mice received 0.2 mg/kg buprenorphine subcutaneously. The sciatic nerve was exposed at the mid-thigh level proximal to the sciatic trifurcation by blunt dissection through the biceps femoris. Three chromic gut ligatures (5/0) were tied loosely around the nerve with approximately 1-mm spacing. The ligatures were tied until they elicited a brief twitch in the hindlimb. The incision was closed in layers.

Inflammatory pain was studied in the zymosan A model. Under brief anesthesia, zymosan A (SigmaAldrich, St Louis, MO, 0.06 mg in 20 mL NaCl) was injected subcutaneously into the plantar side of the left hind paw.

### Quantification and statistical analysis

Cells counts are reported as mean ± SEM. Numbers of experiments (mice and cells) are provided in the figure legends. All behavioral experiments were designed to allow comparisons between two groups: double transgenic (expressing Cre and Dre) vs control (expressing Cre only, Dre only, or neither) mice. Behavioral responses are reported as mean ± SEM. Statistical analysis was performed as follows: group means of double transgenic and control mice for all behavioral tests were compared using a 2-sided unpaired Student’s t-test (spontaneous aversive behavior) or a 2-way repeated measures ANOVA, followed by pairwise comparisons with Sidak adjustment for multiple comparisons (t tests and ANOVA performed with SPSS: IBM Corp. Released 2017. IBM SPSS Statistics for Windows, Version 25.0. Armonk, NY: IBM Corp.). Numbers of experiments (cells or mice) and results of the statistical analysis are provided in the figure legends and in Table S1.

## Data Availability

• Raw data have been deposited at ZENODO.org (https://doi.org/10.5281/zenodo.7648723) • This paper does not report original code • Any additional information required to re-analyze the data reported in this paper is available from the lead contact upon request.

## Supplemental information

Document S1. Figures S1–S9 and Table S1

Document S2. Article plus supplemental information
