# Supplementary material for: c-Maf-positive spinal cord neurons are critical elements of a dorsal horn circuit for mechanical hypersensitivity in neuropathy
Source: Cell Rep. 2023 Mar 21;42(4):112295. doi: 10.1016/j.celrep.2023.112295 (PMC10157139; doi:10.1016/j.celrep.2023.112295)
Supplement: Document S1. Figures S1–S9 and Table S1 [file mmc1.pdf]

Cell Reports, Volume *42*

Supplemental information

c-Maf-positive spinal cord neurons are critical elements of a dorsal horn circuit for mechanical hypersensitivity in neuropathy

Noémie Frezel, Matteo Ranucci, Edmund Foster, Hagen Wende, Pawel Pelczar, Raquel Mendes, Robert P. Ganley, Karolina Werynska, Simon d'Aquin, Camilla Beccarini, Carmen Birchmeier, Hanns Ulrich Zeilhofer, and Hendrik Wildner

Supplemental information

Table S1. (related to Fig. 4-6, S6 and S8): ANOVA results and P values for behavioral analysis

Fig.	mouse line	Viral transgene	test	n (TG)	n (control)	ANOVA
4B	c-Maf^{EX}	hM4Di	von Frey	9	9	F(4,64)=3.499; P=0.012
4C			Hargreaves			F(2.32,37.18)=0.183; P=0.86
4D			Cold			F(4,64)=1.284; P=0.286
4E			Pin prick			F(4,64)=3.189; P=0.025
4F			Brush			F(4,64)=2.467; P=0.054
4G			Rotarod			*t test*: BL: P=0.768; post CNO: P=0.094
4H	c-Maf^{EX}	hM3Dq	von Frey	8	5	F(1.47,44)=6.023; P=0.017
4I			Hargreaves			F(4,32)=0.943; P=0.452
4J			Cold			F(4,44)=9.318; P≤0.000
4K			Pin prick			F(1.87,20.565)=0.189; P=0.815
4L			Brush			F(4,44)=0.551; P=0.699
4M			Biting/liking	8	8	t test: P = 0.0032
4M			Flinching	8	8	t test: P = 0.0157
5C	c-Maf^{EX}	hM4Di+CCI	von Frey	8	7	F(5,65)=5.194; P≤0.000
5D			Pin prick			F(5,65)=4.982; P=0.001
5E			Brush			F(5,65)=0.554; P=0.735
5F	c-Maf^{IN}	Hm3Dq+CCI	von Frey	8	8	F(2.6,36.5)= 1.28; P=0.292
5G			Pin prick			F(5,70)= 3.98; P=0.004
5H			Brush			F(5,70)= 2.5; P=0.038
6F	PV^{IN}	iDTR	von Frey	12	16	F(8,208)=26.469; P<0.000
6G			Hargreaves	11	16	F(8,200)=0.303; P=0.964
6H			Cold	12	16	F(8,208)=2.274; P=0.024
6I			Pin prick	5	4	F(8,56)=1.557; P=0.159
6J			Brush	12	16	F(4.47, 116.2)= 1.274; P=0.282
6K			Biting/liking	8	11	*t test*: P = 0.0014
6K			Flinching	8	11	*t test*: P < 0.0001
S6B	c-Maf^{IN}	hM4Di	von Frey	5	5	F(4,32)=5.266; P=0.002
S6C			Hargreaves			F(4,32)=0.772; P=0.551
S6D			Cold			F(4,32)=0.508; P=0.730
S6E			Pin prick			F(4,32)=0.277; P=0.891
S6F			Brush			F(4,32)=0.294; P=0.880
S6G	c-Maf^{IN}	hM3Dq	von Frey	5	8	F(2.43,28.8)=0.812; P=0.510
S6h			Hargreaves			F(4,44)=2.55; P=0.053

S6I			Cold			F(2.26,24.9)=1.904; P=0.166
S6J			Pin prick			F(4,44)=8.20; P≤0.001
S6K			Brush			F(4,44)=3.21; P=0.04
S8C	c-Maf^{EX}	hM4Di+	von Frey	6	6	F(5,65)=0.762; P=0.581
S8D		ZymosanA	Pin prick			F(0.486,2.243)=2.38; P=0.105
S8E			Brush			F(5,65)=0.271; P=0.927

Supplemental figures

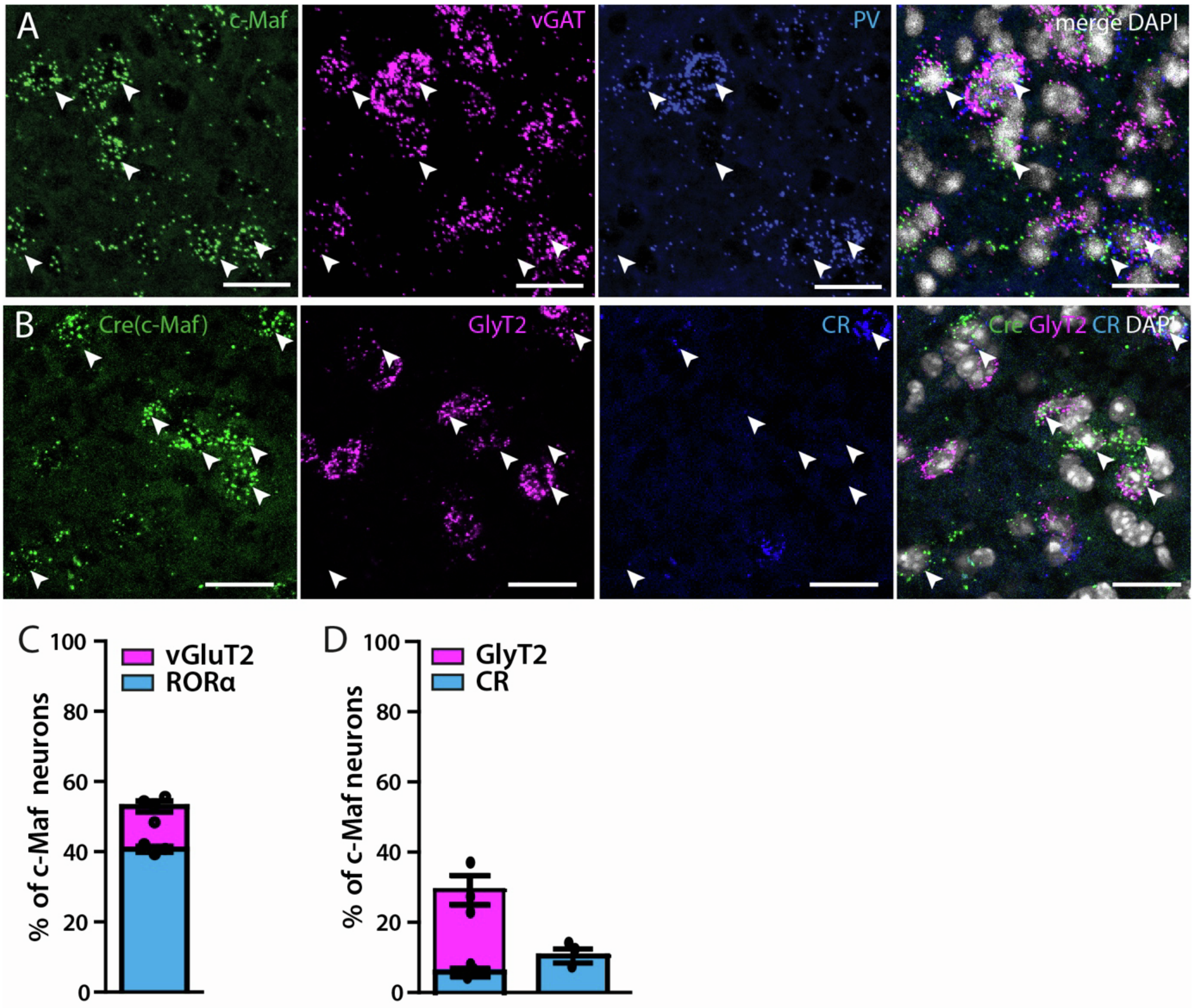

Fig. S1 (related to Fig. 1): ISH showing the proportion c-Maf+ neurons expressing other markers of deep dorsal horn neurons. A. A. Triple ISH showing overlap between c-Maf-, vGat- and PV-expressing neurons. c-Maf^{IN} neurons represent about a third (31.28 ± 1.6%) of all c-Maf neurons and they overlap with inhibitory PV^{+} interneurons (12.75% ± 0.6% of all c-Maf neurons are positive for vGat, i.e. half of the c-Maf^{+}PV^{+} neurons. **B.** Triple ISH showing overlap of Cre (c-Maf)-, GlyT2-and CR-expressing neurons. **C.** Quantification of the overlap between Cre (c-Maf)-, vGluT2-and RORα -expressing neurons (ISH, n = 3 c-Maf^{Cre} mice; 302 Cre^{+} neurons). **D.** Quantification of (C) (n = 3 c-Maf^{Cre} mice; 302 Cre^{+} neurons). **E.** Quantification of the number of c-Maf neurons that are positive for both CCK and PV or both CCK and RORα (n = 4; 878 neurons, and n = 3; 317 neurons respectively). Arrowheads: examples of Cre (c-Maf) positive neurons. Error bars: ± SEM. Scale bars: A: 100 µm, B-C: 20 µm.

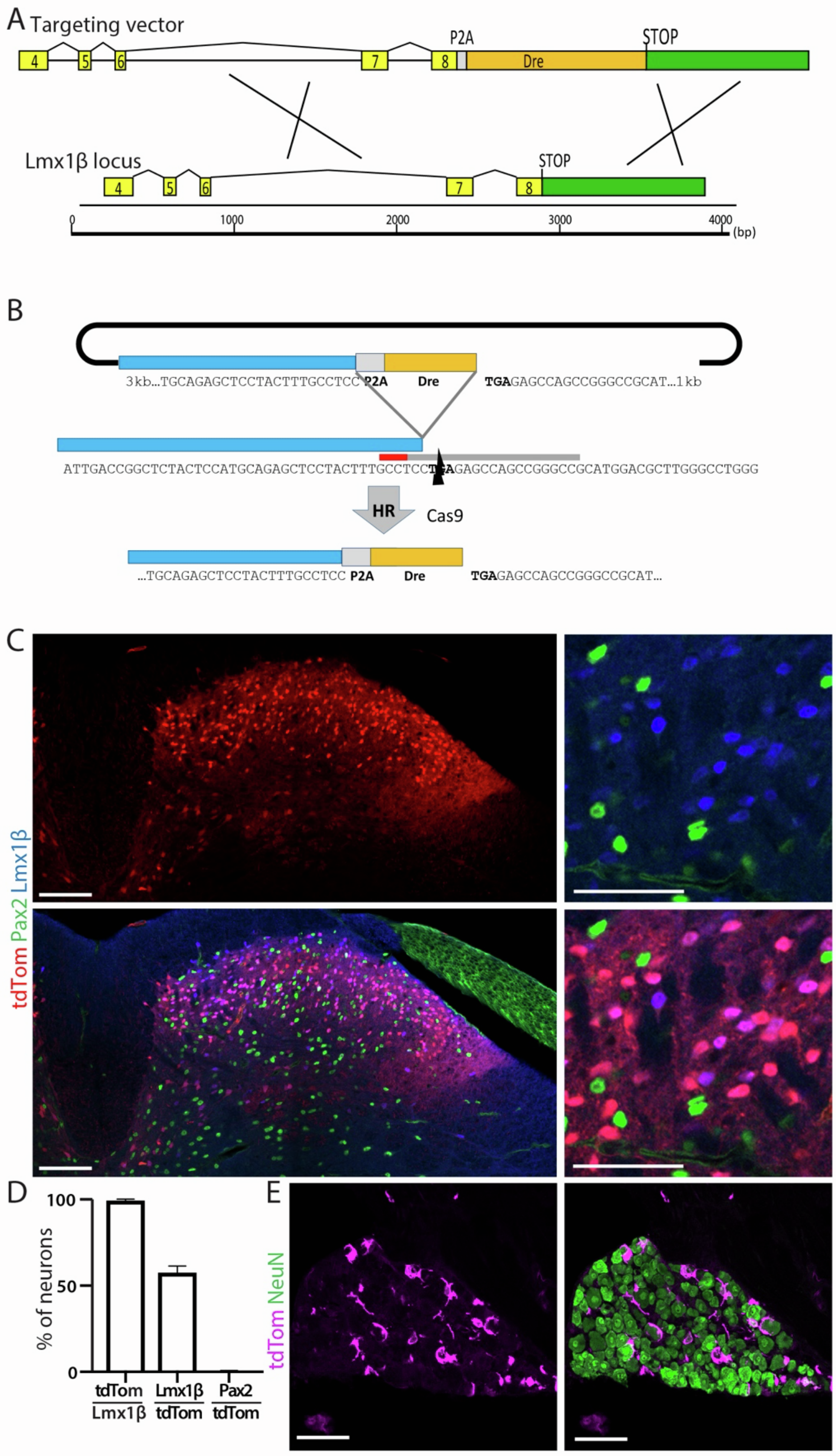

Fig. S2 (related to Fig. 2): Generation of the Lmx1b^{Dre} mouse line. A. Schematic representation of the generation of the Lmx1b^{Dre} allele. A *P2A-Dre* coding sequence was inserted into the STOP codon of the *Lmx1b* gene. **B.** The *Lmx1b* sequence proximal to the STOP codon was scanned using the CRISPOR software (http://crispor.tefor.net/) for the presence of optimal sgRNA target sequences. The target sequence attgtaggagaagac**tca**agagg

residing on the opposite strand and encompassing the *Lmx1b* STOP codon (in bold) was selected and the sgRNA targeting the sequence was transcribed *in vitro* using T7 polymerase (NEB) from a gBlock (IDT) linear DNA template containing the T7 promoter, the Cas9 target sequence and the sgRNA F+E backbone [S1]. An applicable targeting vector was designed to insert the P2A-Dre cassette just upstream of the *Lmx1b* STOP codon with the help of 2.6kb 5' and 1kb 3' homology arms. **C.** IHC showing the overlap between tdTomato, Pax2 and Lmx1b in $Lmx1b^{Dre}$ mice crossed with tdTomato ($Rosa26^{roxStopTom/wt}$) reporter mice. **D.** Quantification of the overlap between tdTomato, Lmx1b and Pax2 in (C) **E.** IHC showing no expression of tdTomato in sensory neurons in $Lmx1b^{Dre}$ mice crossed with tdTomato ($Rosa26^{roxStopTom/wt}$) reporter mice. tdTomato expression in DRGs is due to recombination in satellite glia. Error bars: ± SEM. Scale bars: B: 200 µm, C: 20 µm, E: 100 µm .

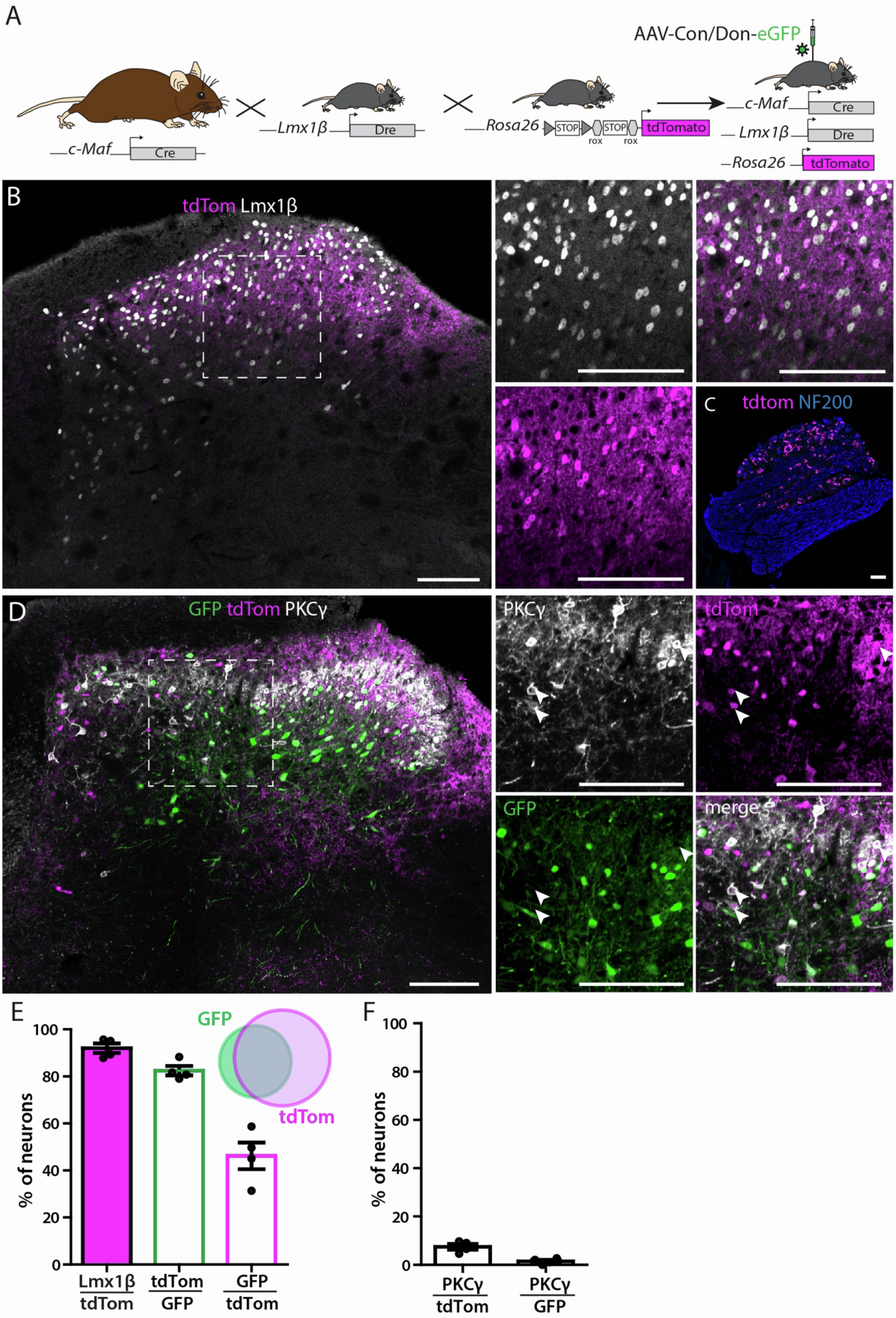
A
AAV-Con/Don-eGFP
c-Maf
Cre
Lmx1β
Dre
Rosa26
STOP
STOP
rox
rox
tdTomato
B
tdTom Lmx1β
C
tdtom NF200
D
GFP tdTom PKCγ
PKCγ
tdTom
GFP
merge
E
% of neurons
GFP
tdTom
Lmx1β
tdTom
tdTom
GFP
GFP
tdTom
F
% of neurons
PKCγ
tdTom
PKCγ
GFP

Fig. S3 (related to Fig. 2): Characterization of c-Maf^{Cre}; Lmx1b^{Dre}*;* Rosa26$^{dstdTom/wt}$ *(*c-Maf^{EX}; Rosa26$^{dstdTom/wt}$ mice). A. Crossing of c-Maf^{Cre} mice to Lmx1b^{Dre} mice, followed by crossing of the double transgenic line to tdTomato (Rosa26$^{dstdTom/wt}$) reporter mice, and intraspinal injection of rAAV9.CAG.C$_{on}$/D$_{on}$.eGFP. **B.** Immunofluorescence staining on a transverse section of lumbar spinal cord of c-Maf^{EX}*;* Rosa26$^{dstdTom/wt}$ *reporter* mice, showing the overlap between tdTom^{+} and Lmx1b^{+} neurons. The general localization of tdTom^{+} neurons in adult mice spinal cord was very similar to that of eGFP^{+} neurons in c-Maf^{EX} mice after viral injection. **C.** Immunofluorescence staining of DRG sections in the same experiment, showing no expression of tdTomato in sensory neurons (n = 4 mice). **D.** Immunofluorescence staining on a transverse section of lumbar spinal cord of c-Maf^{EX}*;* Rosa26$^{dstdTom/wt}$ *reporter* mice injected with rAAV9.CAG.C$_{on}$/D$_{on}$.eGFP showing the overlap between eGFP and tdTom and the location of labelled neurons relative to the PKCγ plexus. **E.** Quantification of the number of tdTom^{+} neurons positive for Lmx1b^{+} in (B) (n = 4; 1523 neurons) and between eGFP^{+} and tdTom^{+} neurons (same samples as in Fig.3C; n = 4 mice; 1523 tdTom^{+} and 853 eGFP^{+} neurons). *The* vast majority of tdTom^{+} neurons expressed Lmx1b (92.03 ± 2.0 % of tdTom^{+} neurons). 82.45 ± 2.03 % of eGFP^{+} neurons expressed tdTom. Conversely, 46.20 ± 5.7% of tdTom^{+} neurons expressed eGFP. **F.** Quantification of the overlap between eGFP^{+} and tdTom^{+} neurons with PKCγ in (D) (n = 4 mice, 571 eGFP^{+} and 857 tdTom^{+} cells). In contrast to eGFP, tdTom was expressed in a few PKCγ^{+} neurons (7.49 ± 1.16%) that had transiently expressed Cre and Dre during development. Arrowheads: examples of PKCγ^{+} tdTom^{+} neurons. Error bars: ± SEM. Scale bars: 100 µm.

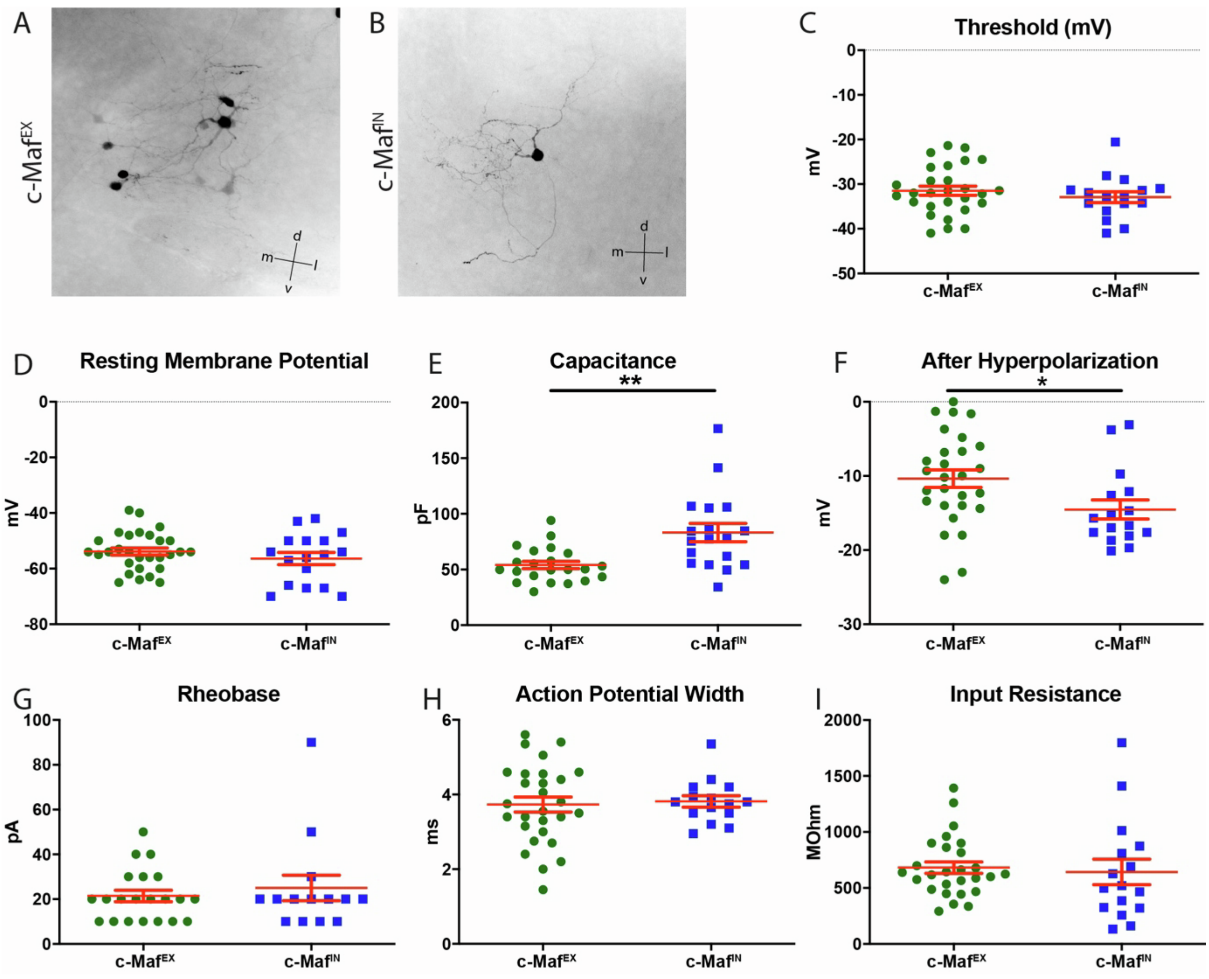

Fig. S4 (related to Fig. 2): Morphological and biophysical characterization of c-Maf^{EX} and c-Maf^{IN} neurons.

A, B. Neuronal morphology of c-Maf neurons revealed by sparse virus mediated labelling. **C-I.** Biophysical characterization of c-Maf^{EX} and c-Maf^{IN} neurons. **C.** Threshold potential, c-Maf^{EX} = -31.46 ± 1.028 mV (n = 28), c-Maf^{IN} = -32.9 ± 1.2 mV (n = 16), unpaired t-test p = 0.4. **D.** Resting membrane potential, c-Maf^{EX} = -53.9 ± 1.3 mV (n = 29), c-Maf^{IN} = -56.4 ± 2.2 mV (n = 17), unpaired t-test p = 0.3. **E.** Capacitance, c-Maf^{EX} = 54 ± 3.3 pF (n = 22) , c-Maf^{IN} = 83.2 ± 8.2 pF (n = 18), unpaired t-test p = 0.001 **F.** After hyperpolarization, c-Maf^{EX} = -10.4 ± 1.2 mV (n = 28), c-Maf^{IN} = -14.5 ± 1.3 mV (n=16), unpaired t-test p = 0.03. **G.** Rheobase, c-Maf^{EX} = 21.4 ± 2.5 pA (n=21), c-Maf^{IN} = 25.0 ± 5.7 pA (n=14), unpaired t-test p = 0.5 **H.** Action potential width, c-Maf^{EX} = 3.7 ± 0.2 ms (n=28), c-Maf^{IN} = 3.8 ± 0.2 ms (n=15), unpaired t-test p = 0.8 **I.** Input resistance, c-Maf^{EX} = 681.9 ± 51.3 MOhm (n=27), c-Maf^{IN} = 642.9 ± 114.1 MOhm (n = 16) unpaired t-test p = 0.7. Error bars: ± SEM.

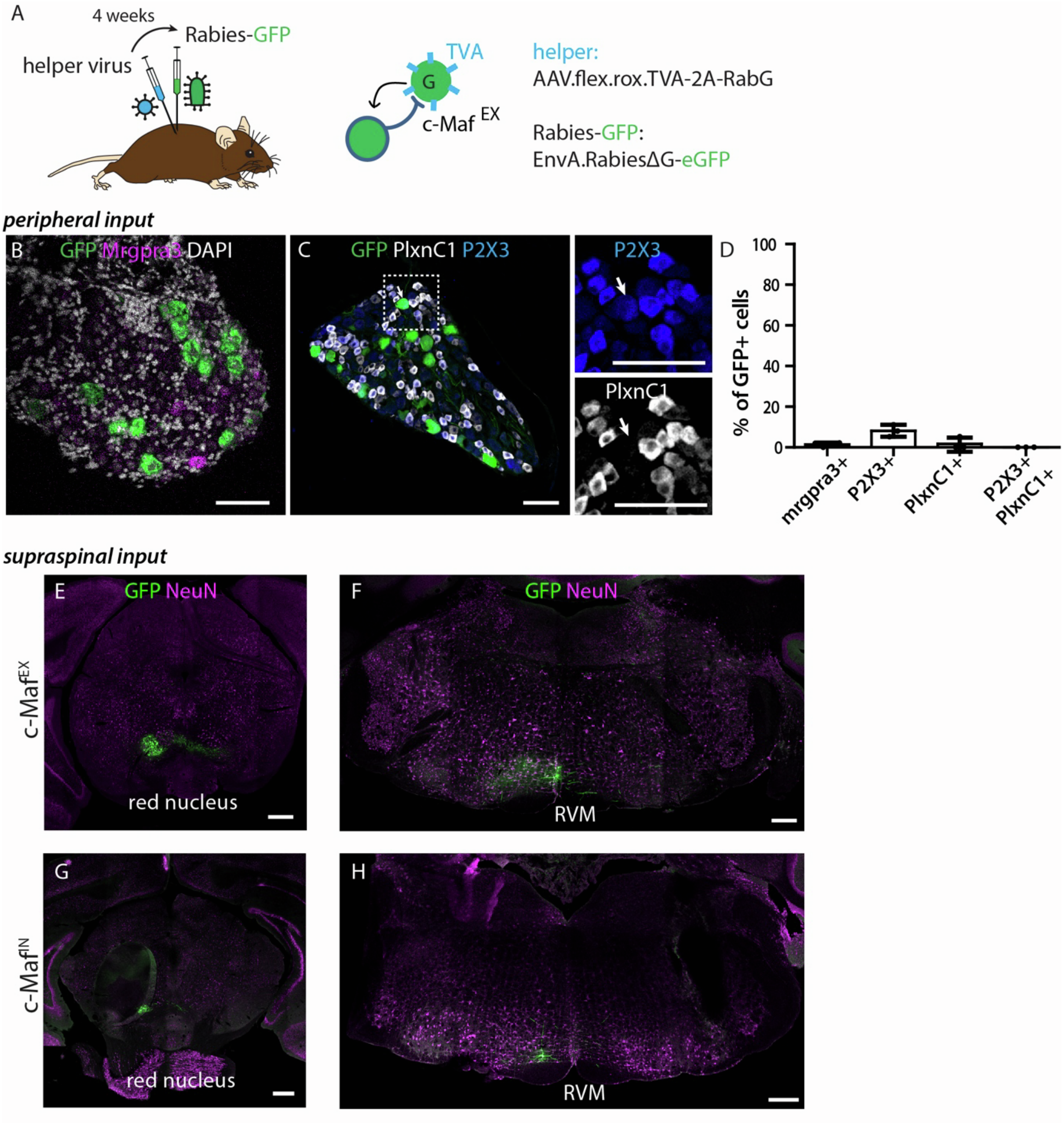

Fig. S5 (related to Fig. 3): Retrogradely labelled sensory neurons after rabies virus-based monosynaptic tracing from c-Maf^{EX} and c-Maf^{IN} neurons. A. A helper virus (TVA, RabG) was injected in the spinal cord of c-Maf^{EX} mice, followed by injection of the EnvA-pseudotyped rabies virus (EnvA.RV.ΔG.eGFP). **B.** Multiplex *in situ* hybridization on DRG sections showing overlap between *eGFP* and *Mrgpra3*. **C.** IHC on DRG sections showing the overlap between eGFP, PlxnC1 and P2X3. Arrow points to a GFP^{+}; P2X3^{+}; PlxnC1^{-} neuron. **D.** Quantification of the number of *eGFP*$^{+}$ DRG neurons positive for *Mrgrpa3* (n = 3, 374 *eGFP*$^{+}$ neurons), P2X3 and PlxnC1 (n = 3 mice, 152 eGFP^{+} neurons). P2X3^{+}Plxnc1^{+} neurons = NP1-2-3 populations [S2]. **E-H.** Immunofluorescence staining showing eGFP labelled neurons in supraspinal sites retrogradely traced from c-Maf^{EX} (I-K) or c-Maf^{IN} (L-N) neurons. Neurons

were found in the red nucleus (RN), and in the rostroventral medulla (RVM) (n = 4). Error bars: ± SEM. Scale bars: 30 µm (B,C) and 100 µm (E-H).

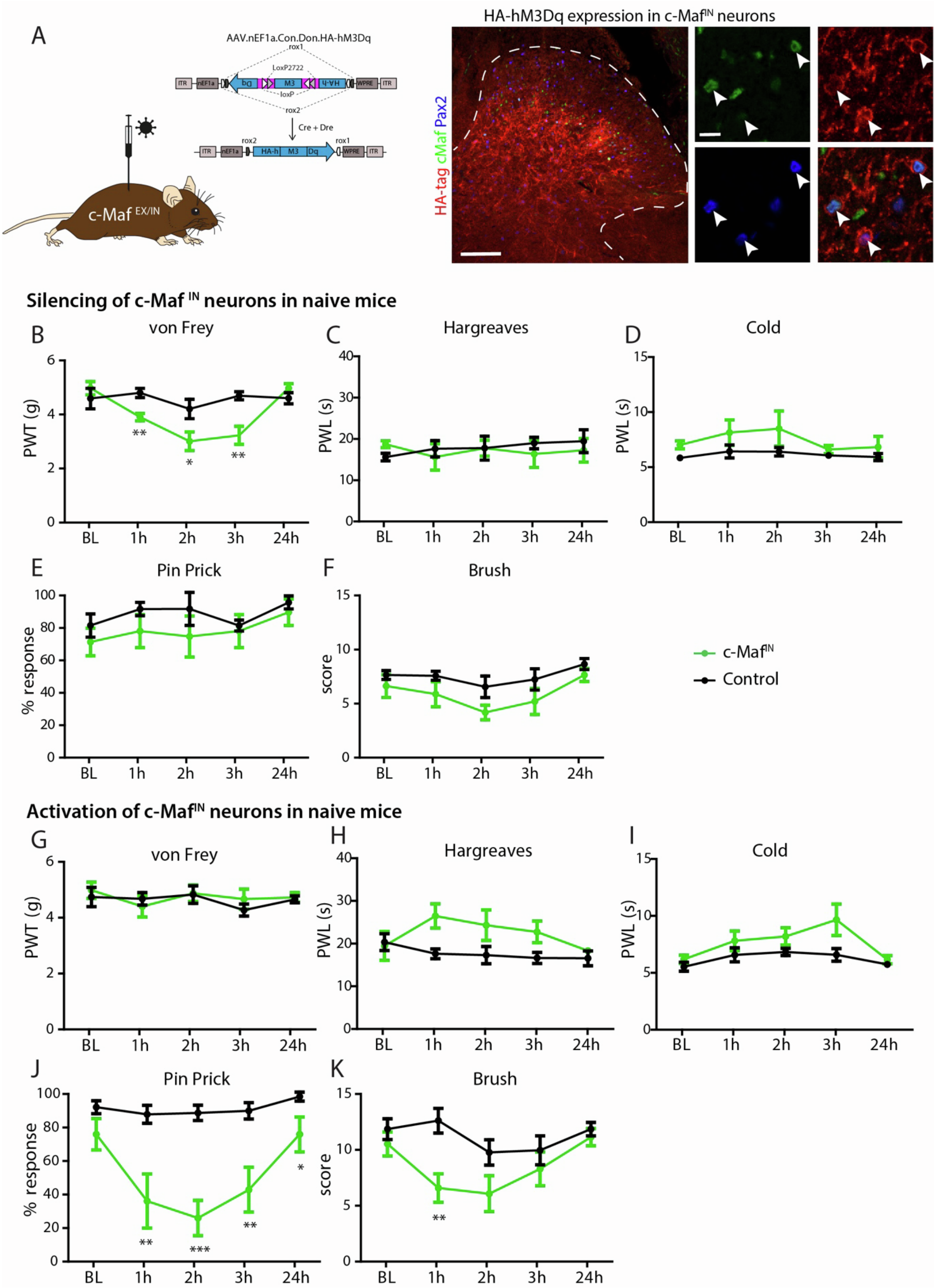

Fig. S6 (related to Fig. 4): Pharmacogenetic silencing and activation of c-Maf^{IN} spinal interneurons in naïve mice. A. Schematic representation of the viral construct used for encoding of Cre-and-Dre dependent transgenes [S3]. Representative image displays DREADD

expression (HA-tag) driven by the AAV.EF1α .C_{on}/D_{on}.HA-hM3Dq injected into the lumbar spinal cord of c-Maf^{IN} mice. **B-F.** Behavioral responses after hM4Di-mediated silencing of c-MafIN neurons (hM4Di: c-MafIN: n = 5; control: n = 5, Table S1). **G-K.** Behavioral responses after hM3Dq-mediated activation of c-Maf^{IN} neurons (hM3Dq: c-Maf^{EX}: n = 5; control: n = 8; Table S1). Activation of c-Maf^{IN} neurons increased sensitivity to mechanical (Pin Prick and brush) stimulation. PWT: paw withdrawal threshold; PWL: paw withdrawal latency; BL: baseline (pre-CNO); 1h to 24h refers to time post CNO injection. Error bars: ± SEM. Scale bars: 100 µm (overview image) and 10 µm (higher magnification images). Number of mice and statistics are shown in Table S1. In brief: * = $p < 0.05$, ** = $p < 0.01$, *** = $p < 0.001$ (ANOVA, followed by pairwise comparisons)

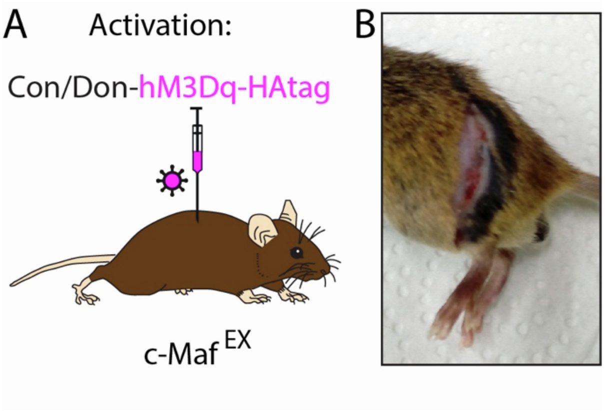

Fig. S7 (related to Fig. 4): Skin lesion induced by scratching and biting of the left flank after repeated CNO injection on mice expressing hM3Dq in c-Maf^{EX}.

A. rAAV.EF1α.C$_{on}$/D$_{on}$.hM3Dq was injected into the lumbar spinal cord of c-Maf^{EX} and control mice. The mice received CNO injections from 14 days after virus injection. **B.** c-Maf^{EX} mice receiving repeated CNO injections also developed skin lesions on the flank ipsilateral to the hM3Dq virus injection over the course of 8-15 days.

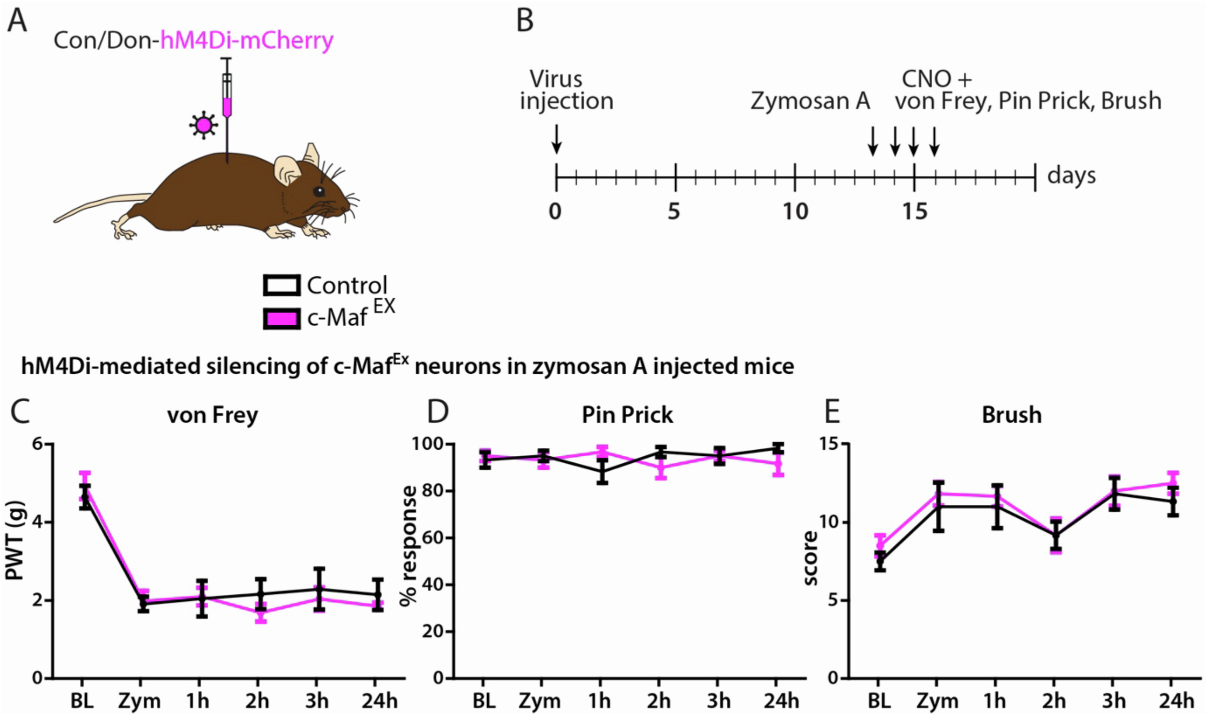

Fig. S8 (related to Fig. 5): Pharmacogenetic Activation of c-Maf^{EX} spinal interneurons in Zymosan A-induced inflammatory pain. A. DREADD expression was driven by injection of rAAV.EF1α.C_{on}/D_{on}.hM3Dq into the lumbar spinal cord of c-Maf^{EX} and control mice. **B.** Virus injection was followed by intraplantar injection of zymosan A to induce inflammatory pain. **C-E:** Responses to mechanical stimulation using the von Frey (C), Pin prick (D) or light brush (E) tests before and after induction of inflammatory pain with zymosan A injection (c-Maf^{EX}: n = 6; control: n = 6, Table 2). PWT: paw withdrawal threshold; BL: baseline before injury; Zym: BL 24 hours after intraplantar injection of zymosan A and before CNO injection; 1h to 24h refers to time post CNO injection. Error bars: ± SEM. Number of mice and statistics are shown in Table 2.

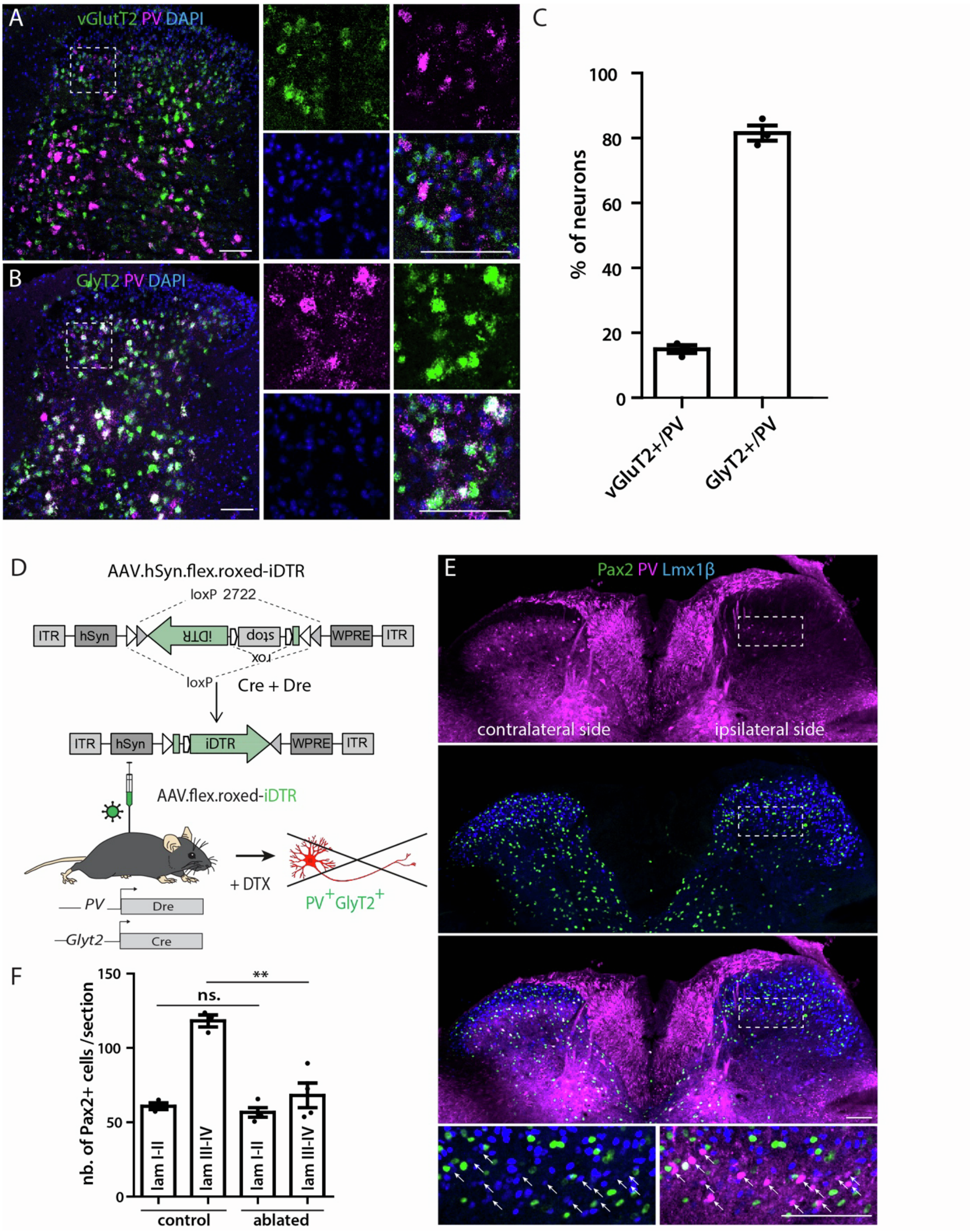

Fig. S9 (related to Fig. 6): Characterization and ablation of PV^{IN} dorsal horn neurons. A. Multiplex *in situ* hybridization showing the overlap between vGluT2$^+$ and *PV*$^+$ neurons in the spinal dorsal horn. **B.** ISH showing the overlap between *GlyT2*$^+$ and *PV*$^+$ in the spinal dorsal horn. **C.** Quantification of (A) and (B) (n = 3; 469 PV+ neurons). The majority of *PV*$^+$ neurons are inhibitory (PV^{IN}) as expected [S4,5] (81.57 ± 2.3% express *GlyT2*), and a smaller

subpopulation is excitatory (14.99 ± 1.2% express *vGluT2*). **D.** PV^{IN} neurons were ablated by injection of a virus carrying a transgene for a Cre-and-Dre dependent iDTR (AAV1.EF1α-flex-rox.iDTR(HB-EGF).hGH) followed by i.p. injection of DTX in GlyT2::Cre; $Pvalb^{Dre}$ double transgenic mice. **E.** IHC showing the ablation of PV^{+} neurons in the dorsal horn. The majority of remaining PV^{+} neurons are excitatory ($Lmx1b^{+}Pax2^{-}$, white arrows in bottom insets). **F.** Quantification of (E) in laminae I-II and III-IV of the dorsal horn (DH) (n = 3 control mice; 608 $Pax2^{+}$ neurons; n = 4 ablated mice; 679 $Pax2^{+}$ neurons). Error bars: ± SEM. Scale bars: 100 μm.

References

S1. Chen, B., Gilbert, L.A., Cimini, B.A., Schnitzbauer, J., Zhang, W., Li, G.W., Park, J., Blackburn, E.H., Weissman, J.S., Qi, L.S., and Huang, B. (2013). Dynamic imaging of genomic loci in living human cells by an optimized CRISPR/Cas system. Cell *155*, 1479-1491. 10.1016/j.cell.2013.12.001.

S2. Usoskin, D., Furlan, A., Islam, S., Abdo, H., Lonnerberg, P., Lou, D., Hjerling-Leffler, J., Haeggstrom, J., Kharchenko, O., Kharchenko, P.V., et al. (2015). Unbiased classification of sensory neuron types by large-scale single-cell RNA sequencing. Nat Neurosci *18*, 145-153. 10.1038/nn.3881.

S3. Fenno, L.E., Mattis, J., Ramakrishnan, C., Hyun, M., Lee, S.Y., He, M., Tucciarone, J., Selimbeyoglu, A., Berndt, A., Grosenick, L., et al. (2014). Targeting cells with single vectors using multiple-feature Boolean logic. Nat Methods *11*, 763-772. 10.1038/nmeth.2996.

S4. Haring, M., Zeisel, A., Hochgerner, H., Rinwa, P., Jakobsson, J.E.T., Lonnerberg, P., La Manno, G., Sharma, N., Borgius, L., Kiehn, O., et al. (2018). Neuronal atlas of the dorsal horn defines its architecture and links sensory input to transcriptional cell types. Nat Neurosci *21*, 869-880. 10.1038/s41593-018-0141-1.

S5. Petitjean, H., Pawlowski, S.A., Fraine, S.L., Sharif, B., Hamad, D., Fatima, T., Berg, J., Brown, C.M., Jan, L.Y., Ribeiro-da-Silva, A., et al. (2015). Dorsal Horn Parvalbumin Neurons Are Gate-Keepers of Touch-Evoked Pain after Nerve Injury. Cell reports *13*, 1246-1257. 10.1016/j.celrep.2015.09.080.
